# Identification of coevolving positions by ancestral reconstruction

**DOI:** 10.1038/s42003-025-07676-x

**Published:** 2025-02-28

**Authors:** Michael G. Nelson, David Talavera

**Affiliations:** Division of Cardiovascular Sciences, School of Medical Sciences, The University of Manchester, Oxford Road, Manchester, UK

**Keywords:** Protein sequence analyses, Coevolution, Molecular evolution, Bioinformatics, Statistical methods

## Abstract

Coevolution within proteins occurs when changes in one position affect the selective pressure in another position to preserve the protein structure or function. The identification of coevolving positions within proteins remains contentious, with most methods disregarding the phylogenetic information. Here, we present a time-efficient approach for detecting coevolving pairs, which is almost perfect in terms of precision and specificity. It is based on maximum parsimony-based ancestral reconstruction followed by the identification of pairs with a depletion on separate changes when compared to their number of concurrent changes. Our analysis of a previously characterised biological dataset shows that the coevolving pairs that we identified tend to be close in the protein sequence and structure, slightly less solvent exposed and have a higher mutation rate. We also show how the ancestral reconstruction can be used to detect favourable and unfavourable amino acid combinations. Altogether, we demonstrate how this approach is essential for identifying pairs of positions with weak covariation patterns.

## Abstract (web summary)

A phylogenetic approach for detecting coevolving pairs via maximum parsimony-based ancestral reconstruction followed by regression analysis of separate and concurrent changes.

## Introduction

**Fig. 1:**
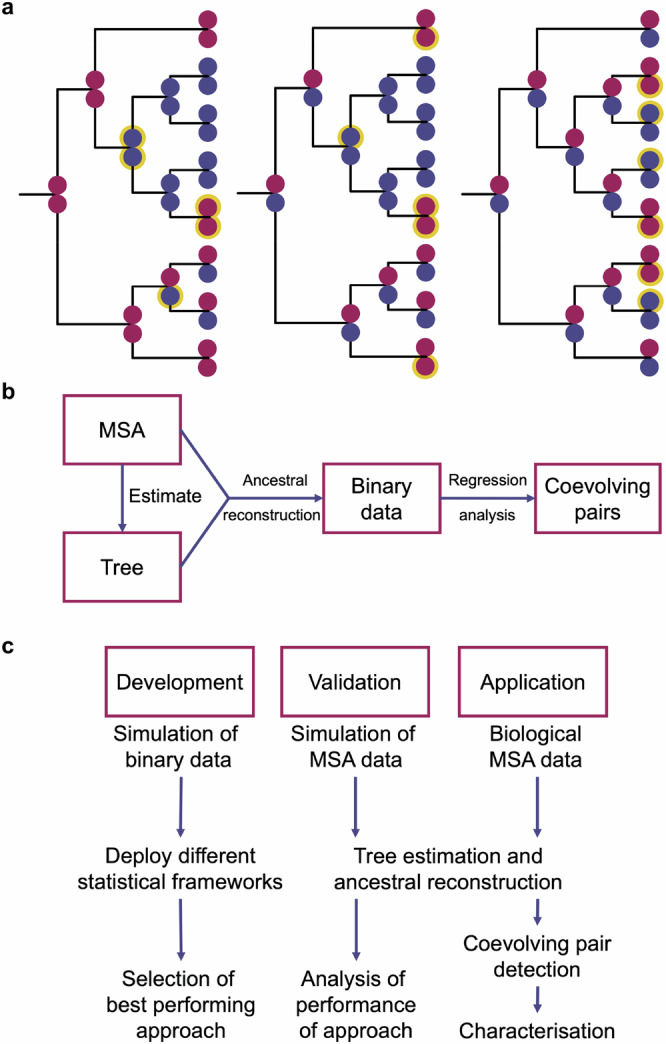
How tree reconstruction affects coevolution detection and the approach to utilise this information. **a** Exemplar phylogenetic trees resulting in the same alignment with different evolutionary histories, two with concurrent changes (left and centre) and one without (right). Pairs of dots represent two example positions from a larger sequence at leaves and internal nodes, red and blue colours indicate the states of each position. Yellow outlined dots highlight positions that have changed at that point in the tree. **b** Schematic of the process to detect coevolving pairs from ancestral reconstruction. **c** Schematic of the approach to method development, validation and application.

It has long been known that some protein positions do not evolve independently^1,2^; the amino acids that they can accommodate depend on other amino acids present in the protein^3–10^. Coevolving positions are essential for the correct folding and function of proteins, and their reliable identification is important in many fields: from protein engineering to genetic diagnostics^11–13^. Since the accurate phylogenetic modelling of molecular coevolution has proven to be very computationally-demanding^14–16^, many tree-independent approaches^17–32^ have been developed in the last decades. These methods identify patterns of covariation within multiple sequence alignments. The challenge faced by these tree-independent methods is how to disentangle the true coevolution-led covariation (i.e. functional or structural constraints) from the spurious covariation caused by the phylogeny (i.e. noise). Different statistical approaches have been developed in order to differentiate these types of covariation^15,33^: e.g., corrected versions of mutual information, or the calculation of a global statistical model for the whole alignment. Several recent studies have used simulated sequence evolution in order to assess the effect of the phylogeny in the covariation signal^34–37^; mutations occurring close to the root of the tree and genetic drift have been identified as causes of spurious covariation. Although not all coevolving residues are close contacts^38^, quite often physical proximity of the predicted residues has been used as a measure of the quality of the predictions^15,28,31,34,35,39^. The rationale is that changes in one residue are assumed to affect the neighbouring residues the most. Some of those tree-independent methods have been hugely successful at predicting pairs of residues physically close in the protein structure^15,28,31^; however, their ability to recognise bona fide coevolution remains controversial (i.e. it is not always clear that the covariation observed in the MSA is the result of molecular coevolution)^37,40^. Moreover, the tree-independent methods do not provide a mechanistic explanation of coevolution^16^. The exemplar evolutionary histories shown in Fig. 1a illustrate how coevolution cannot be determined from covariation patterns alone. Each of those phylogenies result in the same combination of states at the represented positions; however, each tree contains a different number of branches where only one of the positions changes or where both positions change concurrently. Pairs of positions under strong coevolution selection are expected to have a greater proportion of concurrent changes^39,40^. Therefore, one of the main challenges for identifying coevolving positions is to find pairs of positions with an enrichment for concurrent changes.

Here, we present a phylogeny-based approach that allows the identification of likely-coevolving pairs while being less computationally demanding than previous approaches^14^. In order to fulfil this aim, we need to achieve several objectives: 1) to identify the positions in the phylogenetic tree whereby amino acid replacements occurred; 2) to model the accumulation of variation in the absence of covariation; and, 3) to identify pairs of residues whose departure from such a model can be attributed to coevolution (Fig. 1b). We generated synthetic data in order to select and characterise the best identification approach, and we applied the method to the analysis of a previously characterised biological dataset (Fig. 1c).

## Results and Discussion

### Development of approach for detection of coevolving positions

Given a multiple sequence alignment (MSA) and its corresponding phylogenetic tree (MSA-tree duplet), we can count how many concurrent or separate changes occur for each pair of positions in the multiple sequence alignment. Knowing these counts, it is possible to assess if there are more concurrent changes (or fewer separate changes) than expected in a scenario of absence of coevolution.

We use maximum parsimony for performing ancestral reconstruction within a tree; for each position we infer its state at all ancestor nodes, and the branches where changes occur. Subsequently, we count the number of historic separate and concurrent changes. Although maximum parsimony is time-efficient, it might result in some ambiguities; so, we used two slightly different count predictions which we refer to as the branch-level and node-level methods (see Supplementary Information for details).

In the absence of coevolution, the number of times that two positions change concurrently within a single branch of the phylogenetic tree depends solely on their individual probabilities to change. For any given position *i* we can model the number of separate changes with other positions (*S*_*i*_) dependant on the number of concurrent changes with those same positions (*D*_*i*_). Any pair of positions *i* and *j* showing fewer separate changes (*s*_*ij*_) than expected through the model are likely to be coevolving. We tested different statistical frameworks (i.e. linear modelling, generalised linear modelling, and generalised additive modelling) for detecting statistically significant depletion in separate changes on simulated data (Fig. S1). We simulated amino acid replacements considering different rates of evolution, coevolution forces, protein sizes, and alignment depths. Briefly, we simulated 210,000 datasets containing 100 positions and 200 tree splits. Most of the datasets had 0, 1 or 10 covarying pairs each, with covariation strengths –measured as a multiplicative factor- ranging from 2 to 29. These seem realistic scenarios to us based on the finding that we should not expect many observable coevolutionary events^40^. Some datasets (50,000) had a greater number of covarying pairs as we simulated a less likely scenario in which most positions influence each other even if with a weak coevolutionary pressure. Additionally, we assessed the effect of alignment length or depth by simulating 48,000 datasets containing 50–800 positions and 50–102,400 tree splits. Those datasets had a great proportion of covarying pairs with a covariation strength ranging from 2 to 115. See the Methods section and the Supplementary Information for the full details. After modelling the relationship between separate and concurrent changes, we identified outliers.

By modelling scenarios without coevolution, we can assess if any of the approaches is more prone to the selection of false positives. Those results show that neither the generalised linear models nor the Poisson generalised additive model work as well as the other statistical frameworks (Table 1). Scenarios with a specific number of coevolving pairs allow us to assess the ability to detect true positives. Results show that the Negative binomial generalised additive model does not work either (Table 2). Although all linear modelling approaches worked well, a Box-Cox transform was identified as the best performing predictor of coevolving pairs, hence we selected it for the subsequent analyses. It is possible that the slight superiority of the Box-Cox transform over the two other linear modelling approaches relies on the fact that site-specific transforms are used, as opposite to using the same data-transformation approach in all cases. For each pair of positions *λ* is estimated by fitting the ${S}_{\lambda ,i}^{\prime}={\beta }_{0}+{\beta }_{1}{D}_{i}+{\varepsilon }_{i}$ model, and selecting the *λ* that maximises the log-likelihood profile (*λ* value distributions in Fig. S2). The modelling of very different evolutionary scenarios, levels of evolution, alignment depth and length, demonstrates that the linear modelling of the Box-Cox transformed dependent variable results in almost perfect precision and specificity. Therefore, this model will lead to very few false positives. Sensitivity is a more contentious metric in the detection of coevolution^40^. Even so, our approach detects 31.4% of the coevolving pairs in our simulated data. See the Methods and Supplementary Information for a full explanation of the approach, the simulated data and the performance analysis.

**Table 1:** False positives in situation with no coevolution, branches method first, nodes method in brackets

Model	Mean FP per simulation	Median FP per simulation
Linear model	0.142 (0.075)	0 (0)
Logarithmic model	0.060 (0.066)	0 (0)
Box-Cox Transform model	0.013 (0.015)	0 (0)
Poisson Regression model	62.930 (48.142)	36 (25)
Negative binomial regression model	62.143 (47.572)	36 (25)
Poisson GAM	10.267 (6.509)	2 (1)
Negative binomial GAM	1.948 (1.077)	0 (0)

**Table 2:** Performance of different models in situation with one coevolving pair per simulation, branches method first, nodes method in brackets

Model	Precision	Specificity
Linear	0.633 (0.743)	0.999972 (0.999984)
Logarithmic	0.812 (0.793)	0.999988 (0.999987)
Box-Cox	0.945 (0.932)	0.999997 (0.999997)
Negative binomial GAM	0.003 (0.002)	0.999602 (0.999780)

### Validation of approach with synthetic MSA

The Box-Cox transformed model performed the best in our initial synthetic simulations. However, that synthetic data might have been oversimplistic. Given that we used probability distributions to directly generate the binary data, we may have missed some of the apparent incongruities introduced through ancestral reconstruction (e.g., a strongly conserved position being the unique change in a very short branch). For additional validation we applied the same pair-identification approach to synthetic MSA. We performed simulations based on sequence alignments of various depths, artificially evolved with various levels of conservation, without and with selected pairs including a set of three pairs with a common residue, with varied strengths of coevolution using ACES^41^. Background conservation levels were selected in the 60-90% range, comparable to other studies^39^. Phylogenetic trees were estimated from those MSA, and the ancestral reconstruction leading to the binary matrices of changes were performed. Finally, the Box-Cox transformed model was used to identify the coevolving positions.

**Fig. 2:**
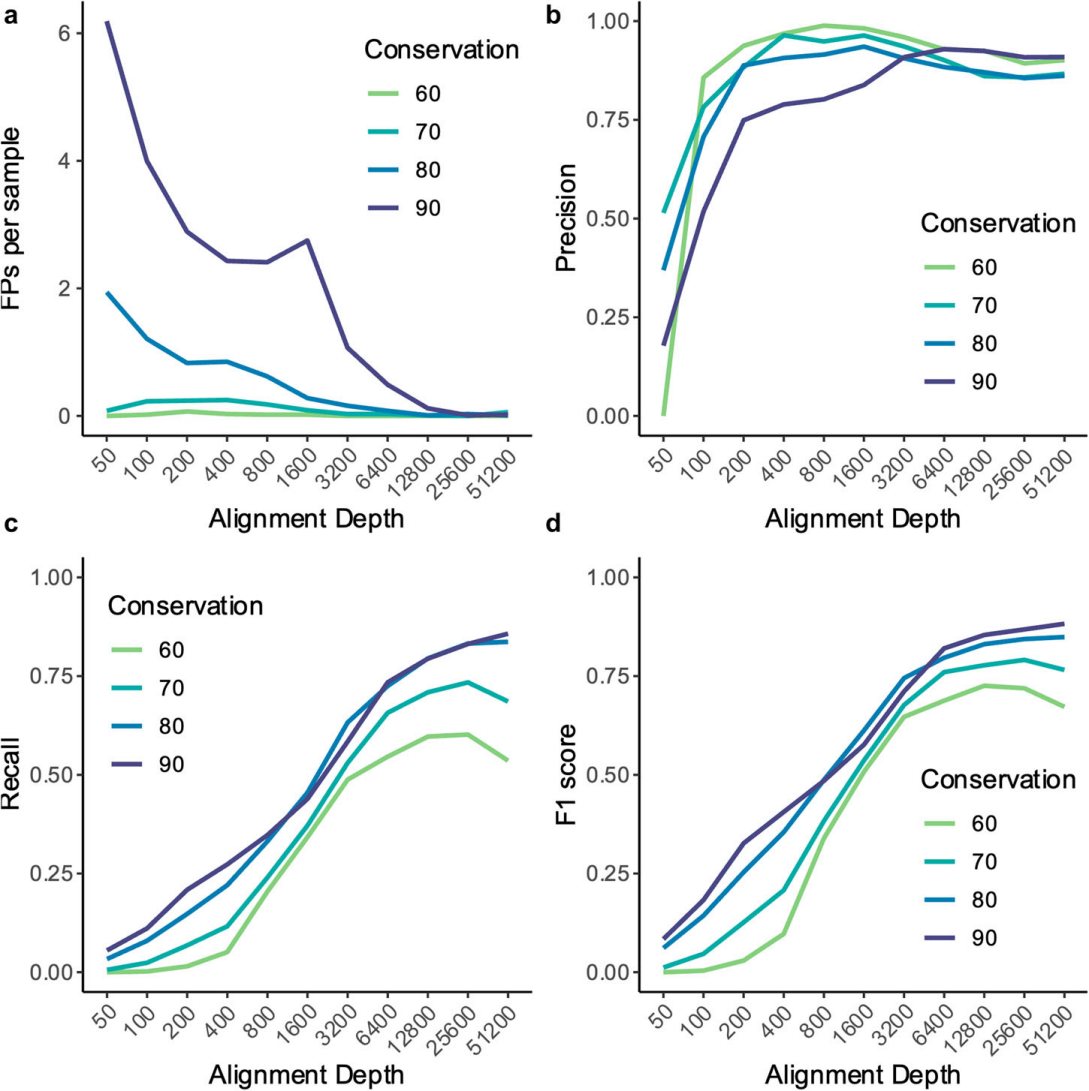
Performance of the method against simulated MSA. Line graph of the mean number of false positive predictions (**a**), precision (**b**), recall (**c**) and F1-score (**d**) per simulated MSA at different conservation level inputs and at various sequence depths for predictions made by both the branches and nodes methods.

Predictions made by both the branches and nodes methods produced very few false positive predictions as in our initial simulations. False positives were observed when conservation was very high or with very small alignments (overlap in Fig. 2a, nodes and branches separately in Fig. S3a and e respectively). In both cases, a small number of changes would be responsible for the apparently spurious coevolution (i.e. would set that pair of positions apart from the position-specific trends). Within the “network” of coevolving pairs the correct connection was selected with a precision of 0.766. Though the incorrect connections within a network were selected more infrequently than the specified coevolving pairs, these false positives made up a significant number of the overall observed false positives (26.4%). Although we categorise these predictions as false positives in this synthetic data, whether these are truly false positives is not clear as, for example residues A + B and A + C coevolving would place an indirect selective pressure between B + C.

Across all simulations with coevolution the approach performs very similarly to our initial testing with average precision of 0.883 and recall of 0.346. On average a recall of over 0.5 is achieved with an alignment depth of 3200 sequences at which depth precision is over 0.9. Recall increases with alignment conservation whereas precision is higher with more diversity coinciding with fewer predictions being made (overlap in Fig. 2b–d, nodes and branches separately in Fig. S3b–d and f–h respectively).

These results confirm that the Box-Cox model performs well not only when applied to synthetic binary data, but also when applied to simulated sequence alignments.

### Application of approach to biological dataset

**Fig. 3:**
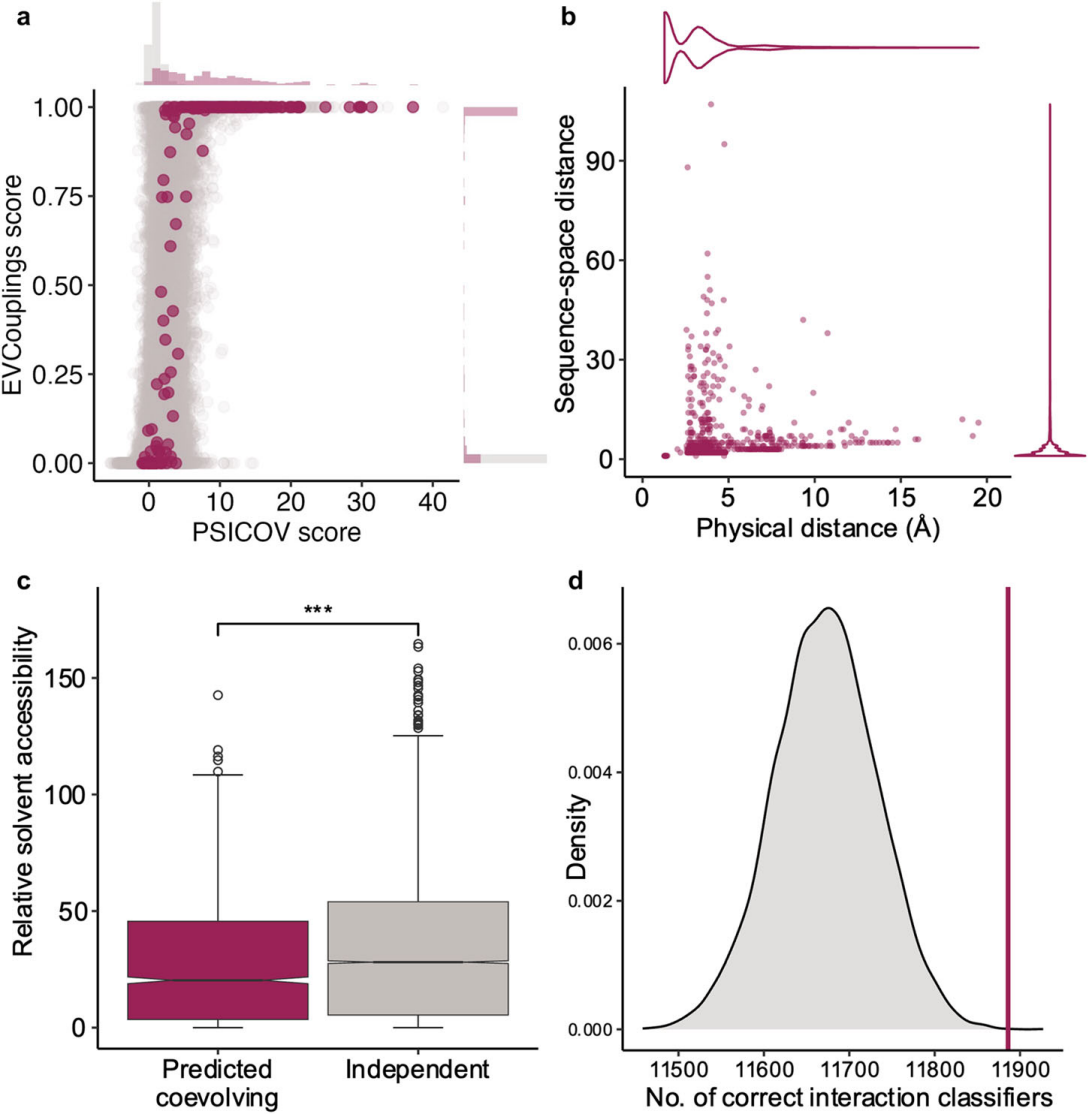
Features of predicted coevolving pairs in the PSICOV dataset. **a** Scatter plot of EVcouplings scores vs. PSICOV scores for all pairs of amino acids greater than 4aa apart. Pairs predicted to be coevolving are coloured red. Distributions of the scores of pairs predicted to be coevolving or not are shown at the top for PSICOV and right for EVcouplings. **b** Physical distance within protein structure versus amino acid distance in sequence for predicted coevolving pairs in the PSICOV dataset. 89.5% of pairs are closer than 5 Å, and 99.7% are closer than 15 Å. **c** Relative solvent accessibility of residues predicted to be coevolving versus all the residues in the PSICOV dataset. **d** Number of times pairs of residues predicted to be coevolving also have favourable structural interactions in comparison with a random selection of pairs.

Then, we obtained and analysed the 150 biological data samples, which had been previously studied in the PSICOV paper^31,40^. The dataset consists of Pfam families with a representative crystal structure, between 50 and 266 residues in length (median 140.5) and with between 1031 and 75,322 sequences (median 3790). We tested the differences that branch-level or node-level reconstruction yield resulting in: 3922 pairs in 131 samples, and 2,551 pairs in 134 samples, respectively (Fig. S4). Running the analyses on 15,000 shuffled datasets resulted in very few false positive predictions (Figs. S5, S6 and, S7). We compared our predicted coevolving pairs with those predicted by two sequence-based tree-independent algorithms: PSICOV^31^ and EVcouplings^42^ (Figs. 3a, S8). The purpose of the analysis was not to compare the accuracy of the predictions; given that we used a biological dataset we did not know for certain which positions were really coevolving. We were interested in assessing to which extent the predictions from phylogeny-free covariation methods and phylogeny-based coevolution methods overlapped. Although PSICOV and EVcouplings have big methodological differences, the comparison of those results to our predicted coevolving pairs yields similar conclusions: 1) half of our predicted pairs result in strong covariation signals that allows identification by those methods; 2) half of the likely-coevolving pairs are not identified by those methods, most likely because of weak covariation signal; and, 3) many covarying pairs selected by those methods do not have strong evidence of coevolution. Some of the pairs in this last group could be due to the expansion of covariation signal of changes occurring near the root of the phylogenic tree^37^. Therefore, examination of the phylogeny is essential for identifying the tendency of positions to change concurrently or independently.

We further analysed the set of 1930 coevolving pairs from 119 protein families, predicted by both the branches and nodes reconstruction methods. Based on our simulated MSAs the precision of those predictions is 0.883. Though pairs are how coevolving residues were detected, they can form networks of interaction (Fig. S9a). The median largest network for a protein was a simple pair (Fig. S9b), though networks could extend to involving 20 residues (Fig. S9b, c) connected by shared coevolution constraints. The majority of coevolving pairs were close in both sequence space (Median = 2 amino acids) and physical distance (Median = 2.917 Å) within the protein structure (Fig. 3b). Whilst not all close residue contacts will result in coevolution and not all coevolving pairs would be expected to be physically close, coevolution between positions close in the protein structure is certainly the simplest to explain. Anishchenko and colleagues found that over 70% of covarying pairs are closer than 5 Å, and just 3% are more distant than 15 Å^38^. Most of the middle-range pairs could be explained by structural variation between homologs or transient alternative conformations, while 35% of the long-range pairs could be explained by homo-oligomerisation^38^. In our results, only three coevolving pairs were physically closer between protein subunits than within them (Fig. S10a). Although there is a significant trend towards lower solvent accessibility for coevolving pairs (Fig. 3c), only 15 out of 119 families showed significant differences between the accessibility of coevolving pairs and that of the rest of the protein (Mann-Whitney test; p-value < 0.05) (Figs. S10b and S11). No enrichment for any specific form of secondary structure (i.e. helix, strand or loop) was observed. Moreover, the number of pairs in the same secondary structure element is not different to the number expected when randomly selecting pairs of positions with the same sequence distance (Fig. S10c). Altogether, these suggest that coevolution selective pressure is more likely to act at the folding stage than at the level of forming the secondary structure.

**Fig. 4:**
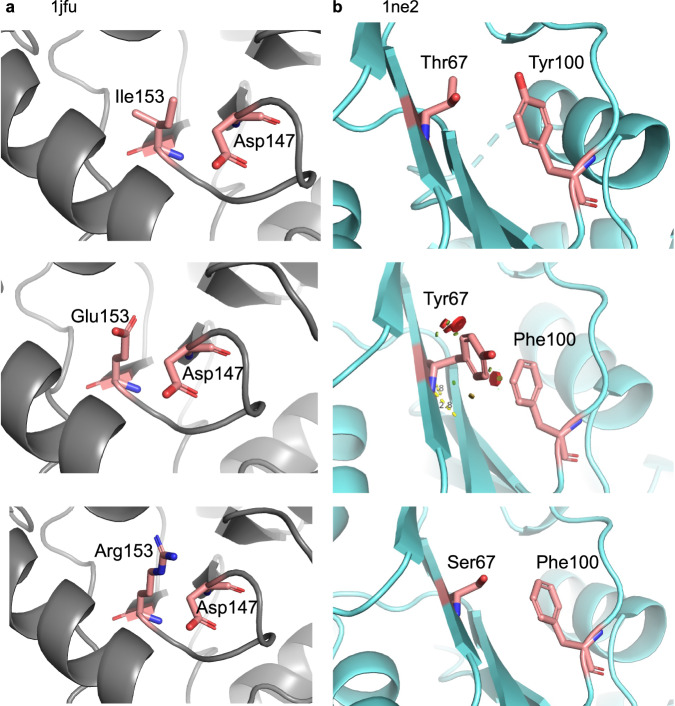
Visual examples of favourable and unfavourable amino acids in coevolving positions. **a** Three panels showing the location of predicted coevolving pair, D147 and I153 in the Redoxin domain (represented by PDB file 1JFU). The top panel is the pair in the crystal structure, middle is a visualisation of a pairing of amino acids under-represented in the tree, possibly due to introducing a charge clash. The bottom panel shows an over-represented pairing possibly due to introducing a favourable charge pair**. b** Three panels showing the location of predicted coevolving pair, T67 and Y100 in the Ribosomal protein L11 methyltransferase (PrmA) domain (represented by PDB file 1NE2). The top panel is the pair in the crystal structure, middle is a visualisation of a pairing of amino acids under-represented in the tree, possibly due to physical implications of two large amino acids introducing a steric clash. The bottom panel shows an over-represented pairing possibly due to maintaining a similar size combination to that observed in the solved structure.

Coevolving positions display higher Shannon entropy than most positions evolving independently (Fig. S10d). This shows that those positions not only have a high mutation rate (Fig. S10e), but also can accommodate multiple amino acids in each position (Fig. S10f). Knowing the location of the changes within the tree is essential in order to identify over-represented and under-represented combinations of amino acids while not overcounting highly-conserved combinations that originated earlier in the phylogeny (Figs. 4 and S12). It should be noted that any combination of hardly observed amino acids it is unlikely to be determined to be statistically under-represented. Assuming that over- and under-representation indicate either favourability or unfavourability of physical interactions between these amino acids, we compared these classifications to previously reported amino acid interaction favourabilities^43^. Over- or under-represented combinations at coevolving positions agreed with favourability classifications significantly more than with random classifiers (Fig. 3d). Although direct physical interaction of residues is not the only reason that we may observe coevolution (e.g., surface charge or polarity balance may also be important)^11,44^, this suggests that selected favourable amino acid pairings are biologically sound.

In conclusion we demonstrated that the use of maximum parsimony for the identification of an excess of concurrent changes within a phylogenetic tree is a powerful method for the detection of coevolving positions. Although some coevolving pairs evolve too slowly to be detected by this approach, many of the identified pairs may have an evolutionary story that make them near-impossible to detect using phylogeny-free approaches. Moreover, coevolution does not only occur in pairs, but can involve groups of residues^6,15,33^; so, the mapping of the changes into the tree may be essential in order to differentiate between true and spurious coevolution^37^. Finally, being less computationally-intensive than maximum-likelihood methods^15^, this or similar approaches may be used in genome-wide studies or in the analysis of massive MSA. Therefore, the research field will extensively benefit from the development of novel phylogeny-based approaches.

## Methods

### Detection of coevolving positions

#### Ancestral reconstruction

Given an MSA-tree duplet, the phylogenetic tree is transversed backwards and forwards in order to infer the tree branches where amino acid replacements occurred. We use ancestral reconstruction based on maximum parsimony, which minimises the number of changes occurring within the phylogenetic tree. Each position (column) in the MSA is treated independently; i.e. its ancestral reconstruction is independent of the other MSA columns. The location of replacements within the tree is stored as binary information (bits) in a two-dimensional matrix: one dimension of the matrix refers to the columns in the MSA, while the other dimension represents the tree splits. See the Supplementary Information for a more comprehensive explanation.

#### Site-specific modelling

For each pair of positions within the MSA (*i* and *j*), the number of separate and concurrent changes (***s***_*i,j*_ and ***d***_*i,j*_, respectively) is counted from that binary-data matrix. We cannot identify an enrichment in concurrent changes just by looking at positions *i* and *j* alone. This is because replacements are not independent of each other –there is only a number of changes that the protein can accommodate and still be the same protein—, and they occur within a tree with different branch lengths –some tree splits will contain more replacements than others. As a consequence, the number of observed concurrent changes is often greater than the expected number based solely on the number of changes occurring in each site. For example, slow-evolving sites are likely to only be replaced in long branches, hence their number of concurrent changes with other sites will tend to be high.

In a protein (MSA) of length *n*, each position *i* has a distribution of values of separate changes (***S***_***i***_ = (*s*_*i,1*_, …, *s*_*i,i-1*_, *s*_*i,i+1*_, …, *s*_*i,n*_)) and a distribution of values of concurrent changes (***D***_***i***_ = (*d*_*i,1*_, …, *d*_*i,i-1*_, *d*_*i,i+1*_, …, *d*_*i,n*_)) with each other position in the MSA. ***S***_***i***_ and ***D***_***i***_ are not independent variables; so, it is possible to model the number of separate changes occurring with other positions dependant on the number of concurrent changes with those same positions (***S***_***i***_ ~ ***D***_***i***_). This site-specific model controls for the non-random distribution of amino acid replacements; by regressing over all the ***S***_***i***_ and ***D***_***i***_ values it models the overall trend of site *i* of having separate or concurrent changes. Sites evolving independently of *i* have their pair of values (***s***_*i,j*_***,d***_*i,j*_) close to the regression line. Outliers with a depletion in separate changes are predicted to be coevolving. See the Supplementary Information for a more comprehensive explanation.

We used 7 different statistical frameworks: 1) linear regression; 2) linear regression of the logarithm-transformed dependent variable; 3) linear regression of the Box-Cox-transformed dependent variable; 4) Poisson-distributed generalised linear modelling; 5) negative binomial-distributed generalised linear modelling; 6) Poisson-distributed generalised additive modelling; and, 7) negative binomial-distributed generalised additive modelling. Linear modelling was achieved by robust fitting of the model; i.e., robust regression using an M estimator. The *rlm()* function from the *MASS* R package^45^ was used to perform the linear modelling. The *glm()* function within the *stats* R package^46^ was use to perform the generalised linear modelling. Generalised additive modelling was performed with the *gam()* function within the *mgcv* R package^47^. We found problems using this latter approach when there were fewer than 10 unique ***D***_***i***_ values. This is because there are fewer unique covariate combinations than maximum degrees of freedom in the smooth function. In order to overcome this issue, the generalised additive modelling approach was restricted to positions that had more than unique 2 ***D***_***i***_ values, and the number of unique ***D***_***i***_ values was used as the dimension of the basis used to represent the smooth term if that number was smaller than 10. This allowed for a large enough basis, while not exceeding the degrees of freedom. Default values were used otherwise. See the Supplementary Information for a more comprehensive explanation.

#### Identification of coevolving positions

After modelling the ***S***_***i***_ ~ ***D***_***i***_ dependency, we estimated which range of ***s***_*i,j*_ values were expected for each ***d***_*i,j*_ value. Coevolving pairs are instances whereby the number of separate changes is smaller than the one expected, given the number of concurrent changes observed between the positions. The *predict()* function within the *stats* R package was used to calculate the prediction intervals in the cases of the linear modelling and generalised additive modelling. In the case of generalised linear modelling we used WenSui Lui’s approach^48^. In order for a pair of positions *i* and *j* to be deemed as a coevolving pair, we required a depletion in separate changes when modelling both ***S***_***i***_ ~ ***D***_***i***_ and ***S***_***j***_ ~ ***D***_***j***_. See the Supplementary Information for a more comprehensive explanation.

### Simulation of synthetic binary datasets

The above-described approach relies on the ability to model the ***S***_***i***_ ~ ***D***_***i***_ dependency and to identify pairs of positions that do not fit with the model. Therefore, the analysis of the binary data matrix is the key step in this process. To test various potential statistical models for assessing coevolution, a comprehensive set of thousands of simulations of binary datasets were performed. These included negative controls simulating independent evolution, datasets with a single coevolving pair, datasets with 10 coevolving pairs and datasets with differing levels of coevolution for every pair in a sequence. In addition, levels of evolution, alignment depth and length were also simulated. Each position in the sequence had a different probability to change, which was drawn from a Beta distribution. Each tree split had a different number of changes, which were derived from a different Beta distribution (multiplied by the length of the sequence). The coevolution strength between pairs of positions was coded by a multiplicative factor; i.e., if one of the positions in the pair was changed in one of the tree splits, the other position had its probability of change multiplied by that factor. Both branches of the tree split had initially the same probability to accumulate changes; however, if one of the coevolving positions was changed, the multiplicative factors was only applied to that branch. We let accumulate changes in each branch of each tree split based on the probabilities and constraints described above. Then, we counted the number of separate and concurrent changes per pair of positions taking into account the specific branches that had changed in each tree split. We also counted the number of changes at the node level; in that case, we only looked at which positions had changed in the tree split, but we did not take into account in which branch the change had occurred. See the Supplementary Information for details on the simulation parameters.

### Assessment of performance

Precision measures the percentage of correct predictions over the number of positive predictions made. This is our metric of choice since we want to maximise the probability that our predictions are true positives. It can also be interpreted as 1 – FDR (False Discovery Rate); so, the higher the precision, the lower the FDR. Specificity measures the percentage of correct predictions over the number of negative cases. Thus, it measures the ability to not misclassify as positives the true negatives. Sensitivity (also known as recall) is not a good metric in our case. The reason is that previous research demonstrated that the number of detectable coevolving pairs that we should expect in a MSA is small^40^; probably, much smaller than the actual number of coevolving pairs. Therefore, we should expect low sensitivity figures. If we tried to maximise the sensitivity hence increasing the number of true positives that we identified, we would likely increase the number of false positives as well. See the Supplementary Information for details on other metrics.

### Simulation of MSAs

To test the performance of the whole approach (from ancestral reconstruction to identification of coevolving positions) on synthetic dataset of MSA, ACES^41^ was used to simulate sequence alignments with varied evolutionary histories. For all simulations all sequences were 130 residues long. Simulations were performed for alignments with depths of 50, 100, 200, 400, 800, 1600, 3200, 6400, 12,800, 25,600 and, 51,200 sequences. Alignments were simulated with 60, 70, 80 or 90% conservation. All alignments were subject to 100% phylogenetic weighting. For independent evolution testing, no coevolving pairs were specified, and each combination of depth and conservation were simulated 100 times. For tests involving coevolution, each alignment contained 27 coevolving pairs, nine each with mutual information (MI) specified as 30, 60 and 90%. Three of these pairs formed a “network” of coevolution made up of four residues, i.e. one position was common to three pairs (residues A + B, A + C and A + D are specified coevolving but not B + C, B + D and C + D). Each combination of depth and conservation were simulated 111 times giving 999 coevolving pairs per MI level. All other settings were left as default. Accompanying phylogenetic trees were produced using FastTree 2.1.11 with the WAG matrix for amino acid substitutions^49,50^. Coevolving pair detection was performed with the Box-Cox statistical framework using both branches and nodes methods and accepting only overlapping predictions.

### Application to a biological dataset

#### Prediction of coevolving positions

To test the coevolving pair detection method on a real biological dataset, the curated subset of 150 Pfam family alignments produced with the jackhmmer program from HMMER 3.0^51^ included in the PSICOV method paper^31^ was utilised (Fig. S13a–c). Accompanying phylogenetic trees were produced using FastTree 2.1.11 with the WAG matrix for amino acid substitutions^49,50^. To analyse performance against a matched dataset with no coevolution (i.e. the same number of sequences per alignment, the same number of positions per alignment, and the same amino acid composition per position in the MSA), new MSAs were created by randomly shuffling all characters (amino acids and gaps) in each column in the protein alignment. Each column was shuffled independently. This process removes the within sequence connection between positions while maintaining the amino acid frequencies within the alignment. Any coevolution signal within these new alignments will be spurious. We generated 100 new shuffled alignments for each of the 150 PSICOV dataset alignments. New trees were created using FastTree to generate 15,000 new alignment-tree duplets. Coevolving pair detection was performed using both branches and nodes methods with the Box-Cox statistical framework for the 150 PSICOV dataset families and 15,000 shuffled families. The first factor that was observed was the high number of predicted pairs containing gaps at the ends of the alignments. Since these generally represent low information locations a filter was applied to remove any predictions where more than 20% of the amino acid combinations in the alignment contained a gap. This mostly occurred at the end of alignments and removed a bias towards predicted coevolving pairs occurring at the ends of proteins (Fig. S13d). This filtering was performed on both the real and shuffled dataset results.

#### Characterisation of coevolving positions

Physical distance was calculated as the closest distance between non-Hydrogen atoms of two residues.

Solvent accessibility was calculated with Naccess (S.J. Hubbard and J.M. Thornton, University College London) and the relative solvent accessibility was used.

Annotation of secondary structure components was extracted from .cif files from PDB. The secondary structure of the coevolving pairs was compared to that of 10,000 random samples matched to 1) the number of predictions per protein and 2) the sequence space distance. Residues were considered in the same secondary structure if they were in the same helix, sheet or linker region.

Shannon entropy was calculated for each position in the alignments using the 22-letter alphabet with the entropy package from bio3d^52^.

For each predicted coevolving pair, a Fisher exact test was performed on the contingency table of ancestral amino acid counts to determine whether the amino acids at the first and second position were independent. For coevolving pairs with a significant (*P* < 0.05), the Bonferroni-adjusted P-values for each amino acid combination were calculated using the standardised χ^2^ residuals^53^.

Over and under-representation of amino acid pairings were compared with structurally favourable and unfavourable combinations, as determined by Betts and colleagues^19^. 10,000 simulated datasets of random agreement or disagreement with favourability were created to assess the chance of random agreement.

EVcouplings was run in monomer mode using the existing alignments from the PSICOV dataset. All other parameters were left as default. PSICOV scores were obtained from the original paper^31^. Comparisons with PSICOV were limited to those predicted coevolving pairs that were greater than 4aa apart in sequence space as these are the residues considered by PSICOV.

### Reporting summary

Further information on research design is available in the Nature Portfolio Reporting Summary linked to this article.

## Supplementary information

Supplementary Information

Reporting Summary

## Data Availability

Code/scripts to generate binary data matrices and scripts to analyse them as well some example data are provided at 10.5281/zenodo.14442667^54^.
