# Supplementary material for: Identification of coevolving positions by ancestral reconstruction
Source: Commun Biol. 2025 Feb 28;8:329. doi: 10.1038/s42003-025-07676-x (PMC11871020; doi:10.1038/s42003-025-07676-x)
Supplement: Supplementary file 1 — Supplementary Information [file 42003_2025_7676_MOESM1_ESM.pdf]

Supplementary Information

for

Identification of Coevolving Positions by Ancestral Reconstruction

by

Michael G Nelson & David Talavera

Table of Contents

- Supplementary Figures S1-S13 …………………….……………... 3
- Supplementary information about method development ………… 25

Supplementary Figures

for

Identification of Coevolving Positions by Ancestral Reconstruction

Supplementary Figure 1. Examples of statistical models of separate and concurrent changes highlighting outliers

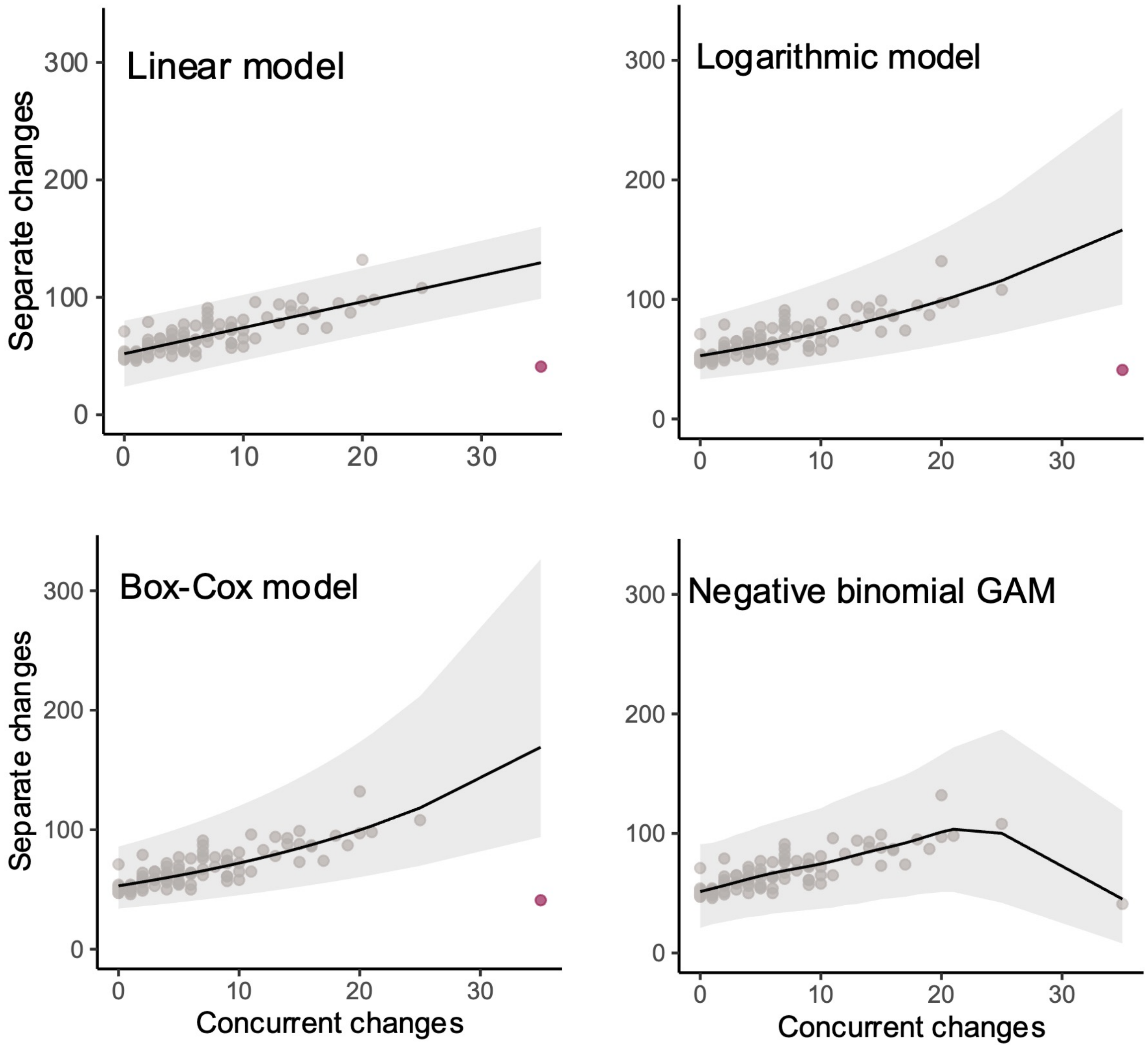

Illustration of the models created for relationship between separate and concurrent changes for a single residue with all other residues in a simulated sequence. Upper and lower confidence values are within the highlighted area and predicted coevolving pairs are coloured red.

Supplementary Figure 2. Box-Cox lambda values for synthetic binary matrix simulations

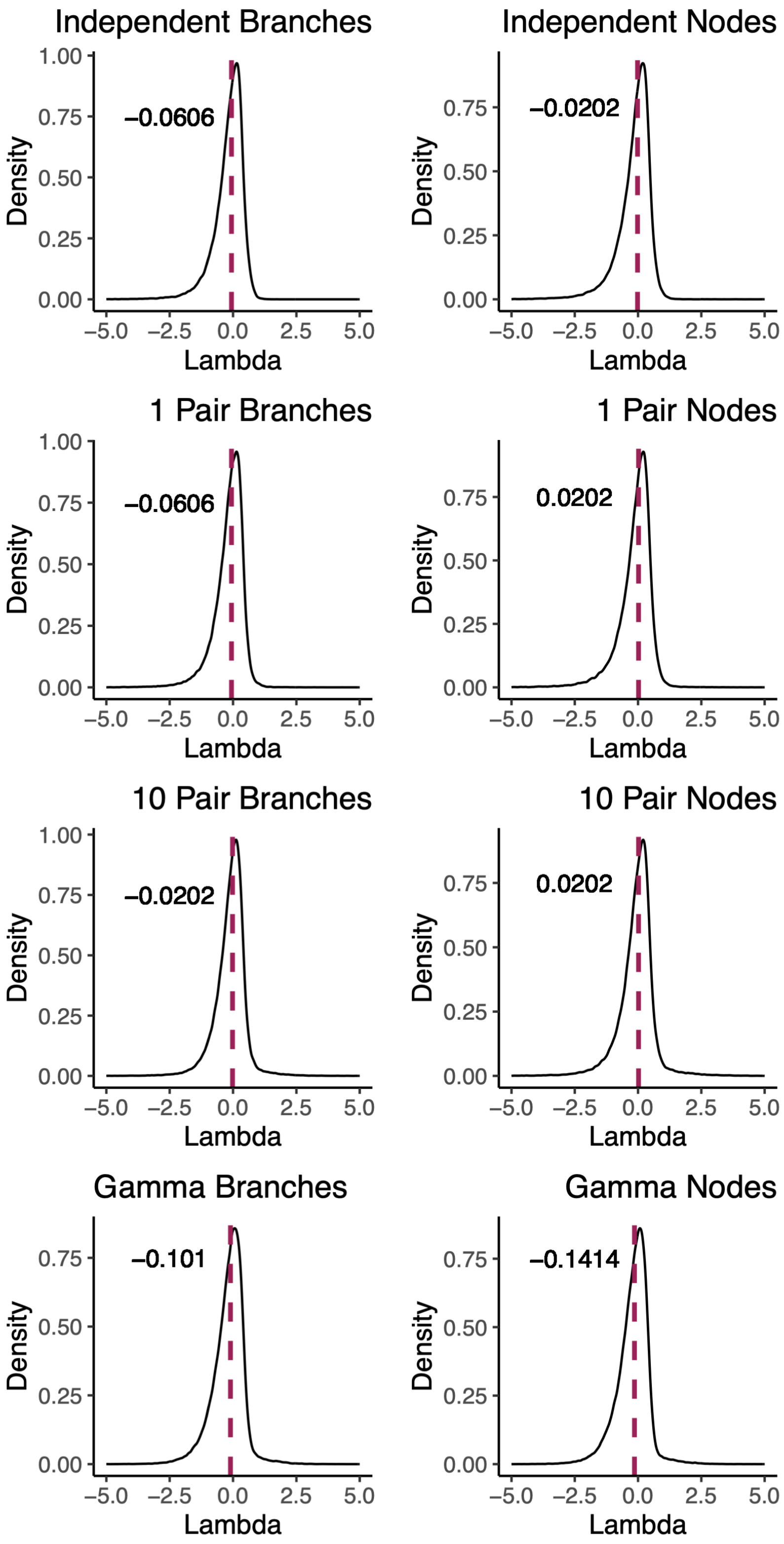

Distributions of Box-Cox λ values from a randomly sampled subset of 1000 simulations from the full sets of simulated data used to assess the models. The median value is illustrated with a dashed line and the value labelled.

Supplementary Figure 3. Performance of the method against simulated MSA

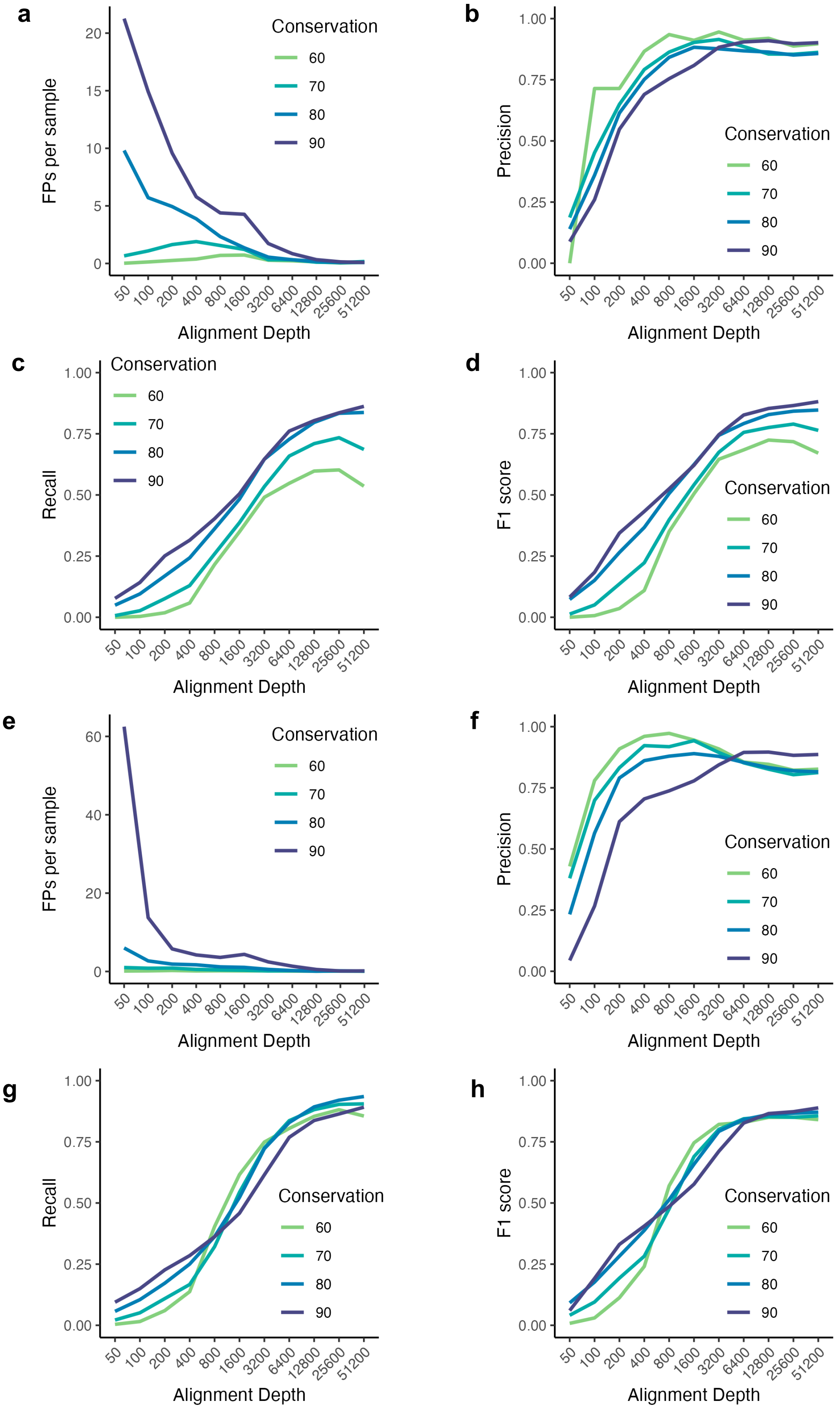

Line graph of the mean number of false positive predictions (**a**), precision (**b**), recall (**c**) and F1-score (**d**) per simulated MSA at different conservation level inputs and at various sequence depths for predictions made by the nodes method. **e-f.** as a-b but for predictions made by the branches method.

Supplementary Figure 4. PSICOV dataset coevolving pair predictions

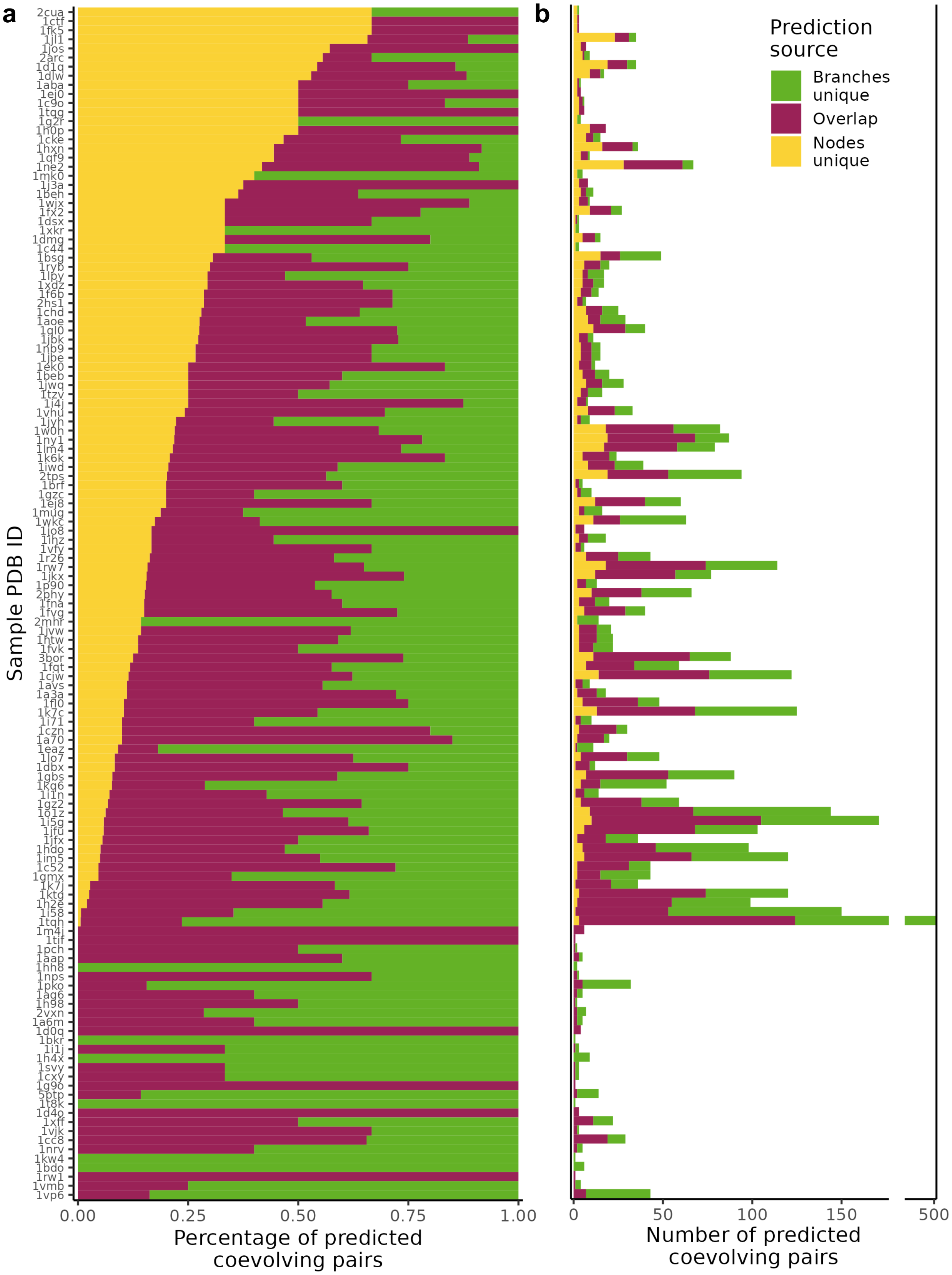

Percentage of predicted coevolving pairs made by the branches and nodes methods and the overlap between the two for each PSICOV sample (**a**). Raw numbers of predictions per sample and their prediction method (**b**).

Supplementary Figure 5. Numbers of coevolving pairs predicted per sample in the PSICOV curated dataset

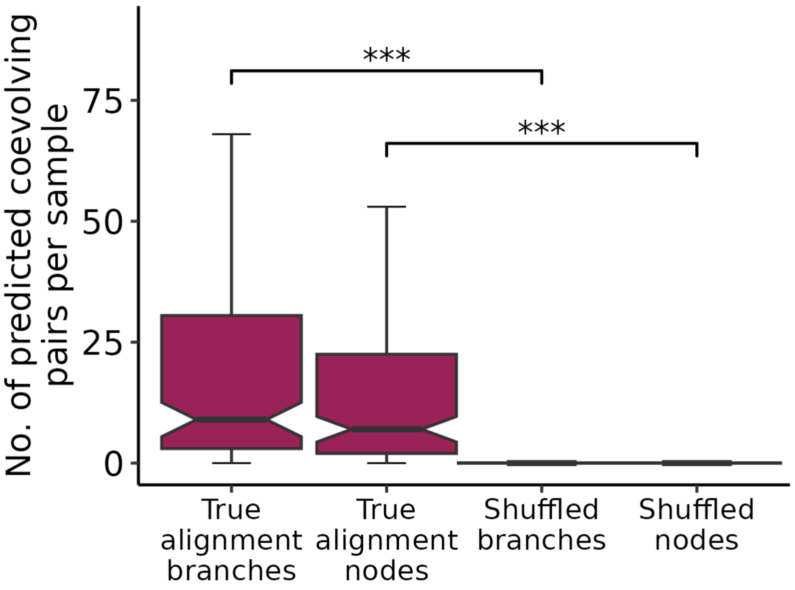

Numbers of coevolving pairs predicted per sample in the PSICOV curated dataset using the Box-Cox model with both the branches and nodes methods versus the numbers predicted in the 15,000 shuffled simulations. (Detailed per sample in Figs S6 and S7).

Supplementary Figure 6. Individual sample coevolving pairs from the branches method

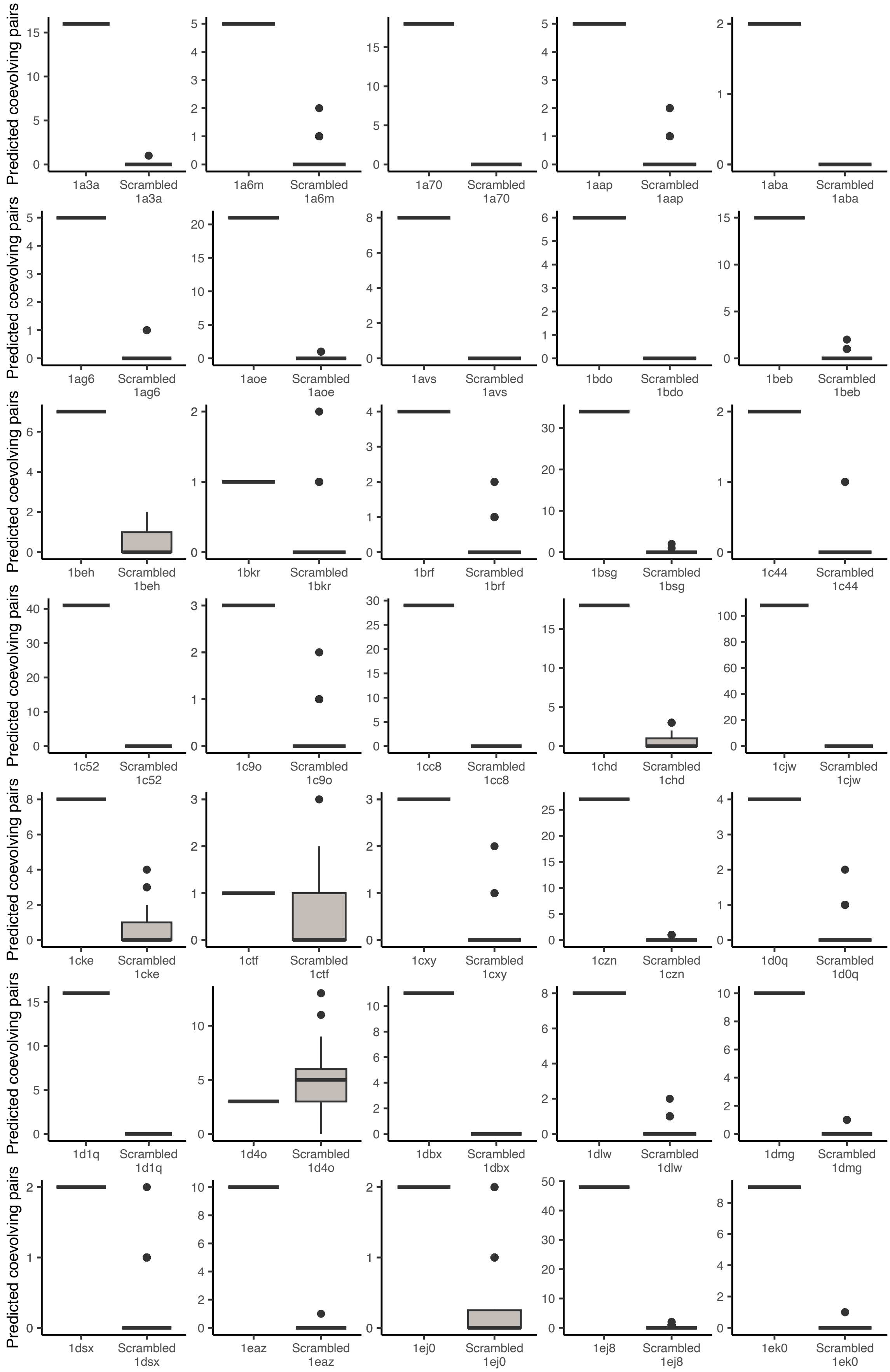

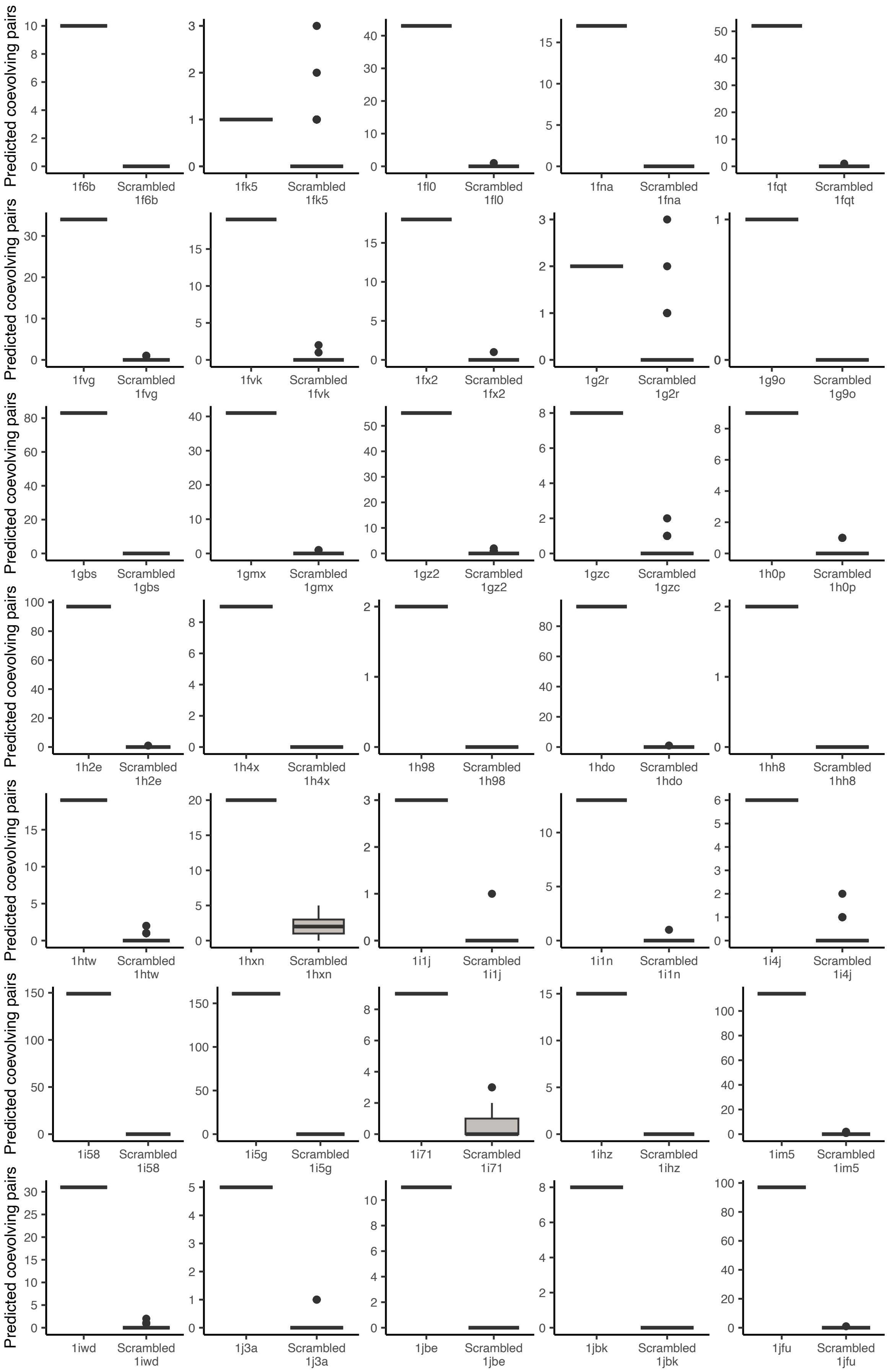

Predicted coevolving pairs
1f6b
Scrambled 1f6b
1fk5
Scrambled 1fk5
1fl0
Scrambled 1fl0
1fna
Scrambled 1fna
1fqt
Scrambled 1fqt
1fvg
Scrambled 1fvg
1fvk
Scrambled 1fvk
1fx2
Scrambled 1fx2
1g2r
Scrambled 1g2r
1g9o
Scrambled 1g9o
1gbs
Scrambled 1gbs
1gmx
Scrambled 1gmx
1gz2
Scrambled 1gz2
1gzc
Scrambled 1gzc
1h0p
Scrambled 1h0p
1h2e
Scrambled 1h2e
1h4x
Scrambled 1h4x
1h98
Scrambled 1h98
1hdo
Scrambled 1hdo
1hh8
Scrambled 1hh8
1htw
Scrambled 1htw
1hxn
Scrambled 1hxn
1i1j
Scrambled 1i1j
1i1n
Scrambled 1i1n
1i4j
Scrambled 1i4j
1i58
Scrambled 1i58
1i5g
Scrambled 1i5g
1i71
Scrambled 1i71
1ihz
Scrambled 1ihz
1im5
Scrambled 1im5
1iwd
Scrambled 1iwd
1j3a
Scrambled 1j3a
1jbe
Scrambled 1jbe
1jbk
Scrambled 1jbk
1jfu
Scrambled 1jfu

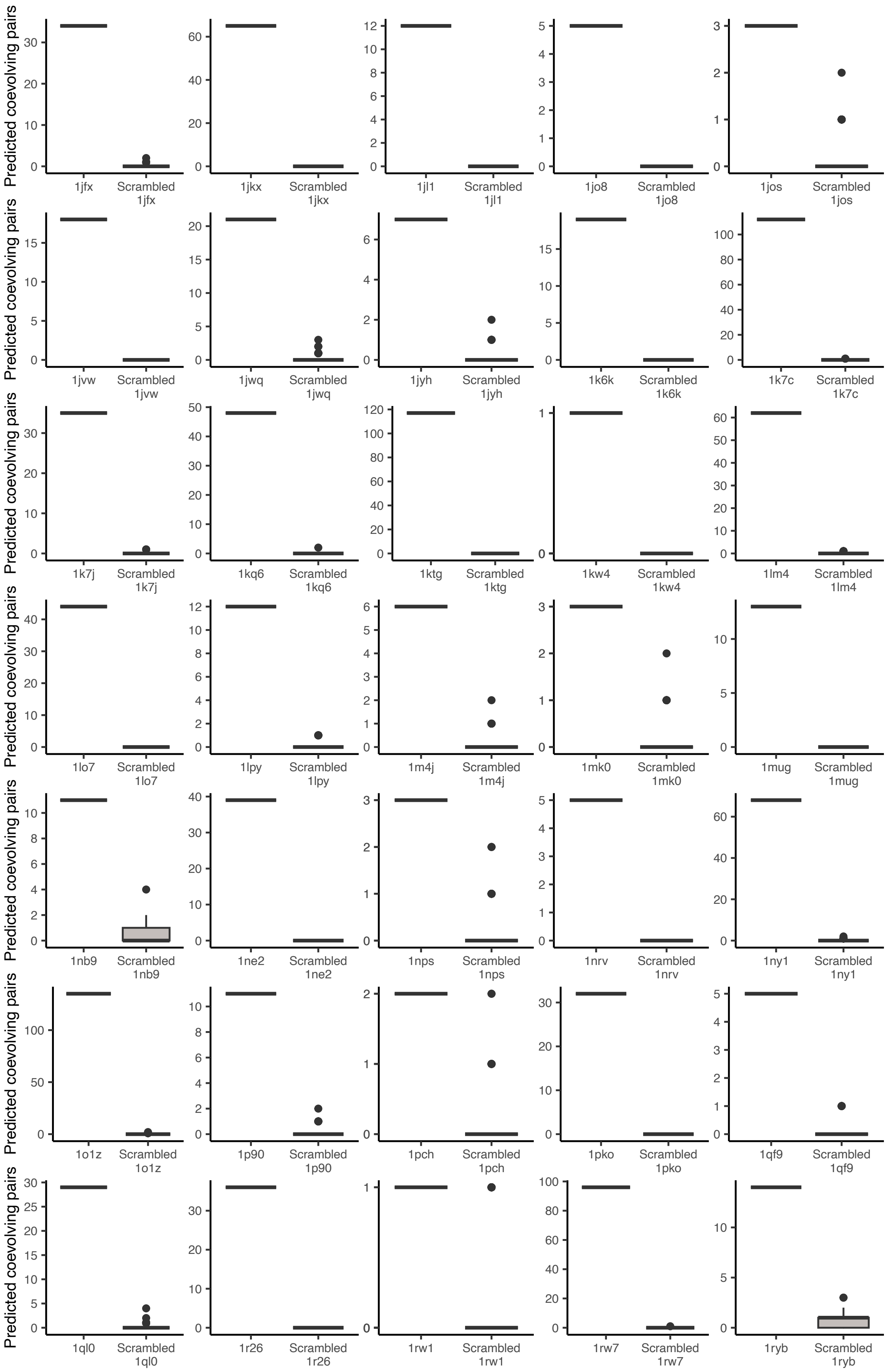
Predicted coevolving pairs
1jfx
Scrambled 1jfx
1jkx
Scrambled 1jkx
1jl1
Scrambled 1jl1
1jo8
Scrambled 1jo8
1jos
Scrambled 1jos
1jvw
Scrambled 1jvw
1jwq
Scrambled 1jwq
1jyh
Scrambled 1jyh
1k6k
Scrambled 1k6k
1k7c
Scrambled 1k7c
1k7j
Scrambled 1k7j
1kq6
Scrambled 1kq6
1ktg
Scrambled 1ktg
1kw4
Scrambled 1kw4
1lm4
Scrambled 1lm4
1lo7
Scrambled 1lo7
1lpy
Scrambled 1lpy
1m4j
Scrambled 1m4j
1mk0
Scrambled 1mk0
1mug
Scrambled 1mug
1nb9
Scrambled 1nb9
1ne2
Scrambled 1ne2
1nps
Scrambled 1nps
1nrv
Scrambled 1nrv
1ny1
Scrambled 1ny1
1o1z
Scrambled 1o1z
1p90
Scrambled 1p90
1pch
Scrambled 1pch
1pko
Scrambled 1pko
1qf9
Scrambled 1qf9
1ql0
Scrambled 1ql0
1r26
Scrambled 1r26
1rw1
Scrambled 1rw1
1rw7
Scrambled 1rw7
1ryb
Scrambled 1ryb

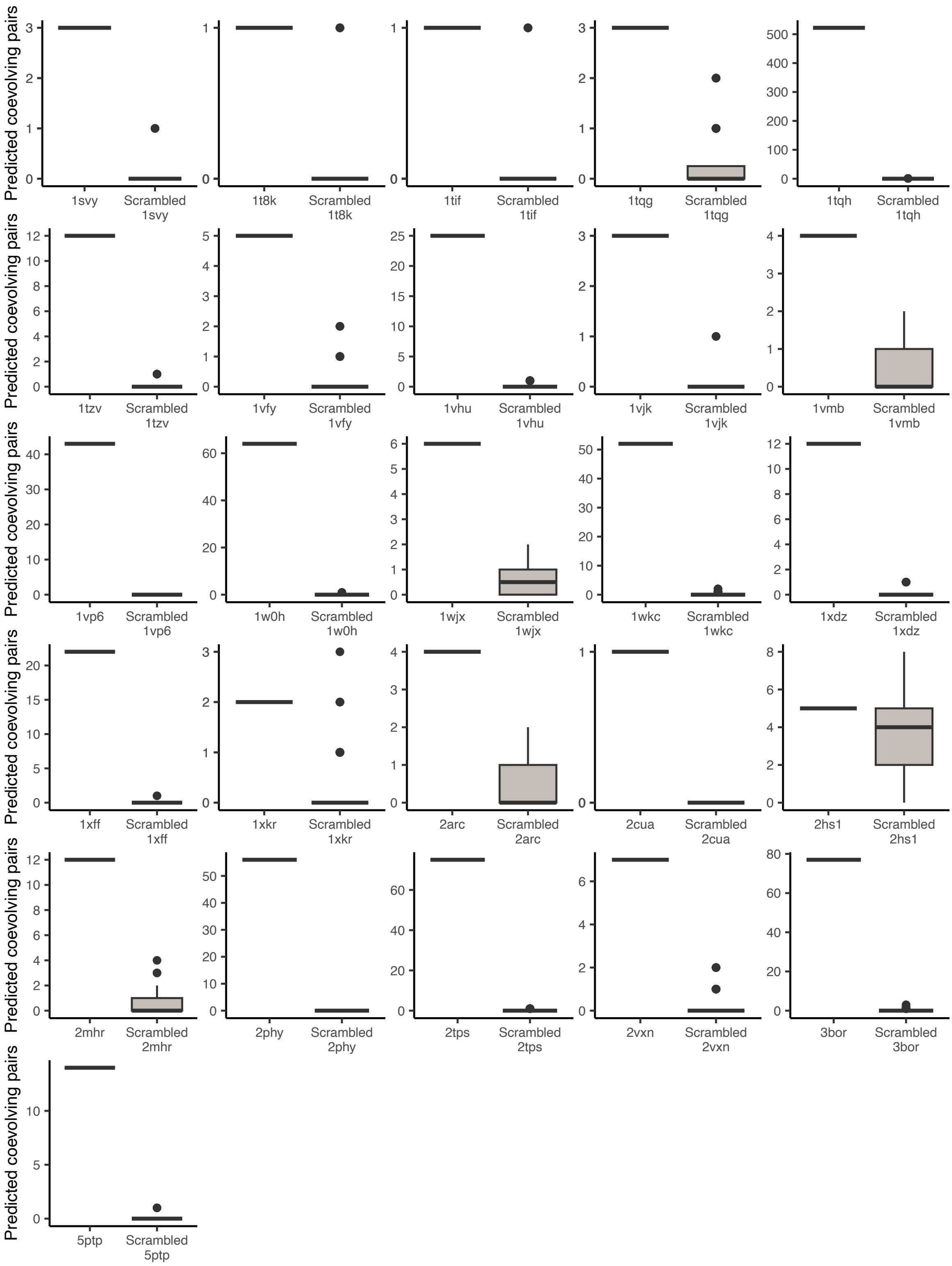

The number of predicted coevolving pairs predicted using the Box-Cox model and branches method in each PSICOV sample versus the numbers predicted in 100 shuffled versions of the alignment.

Supplementary Figure 7. Individual sample coevolving pairs from the nodes method

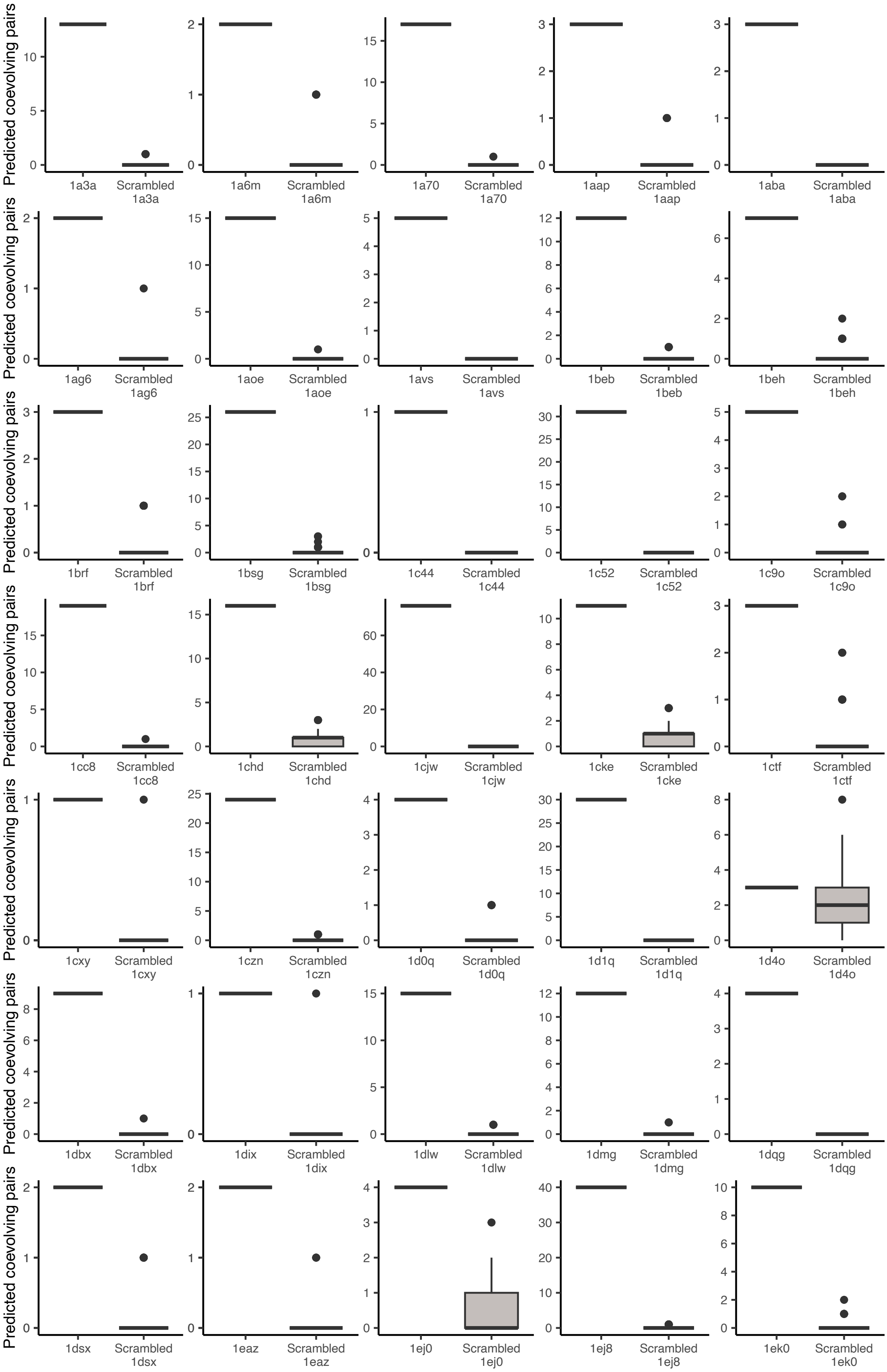

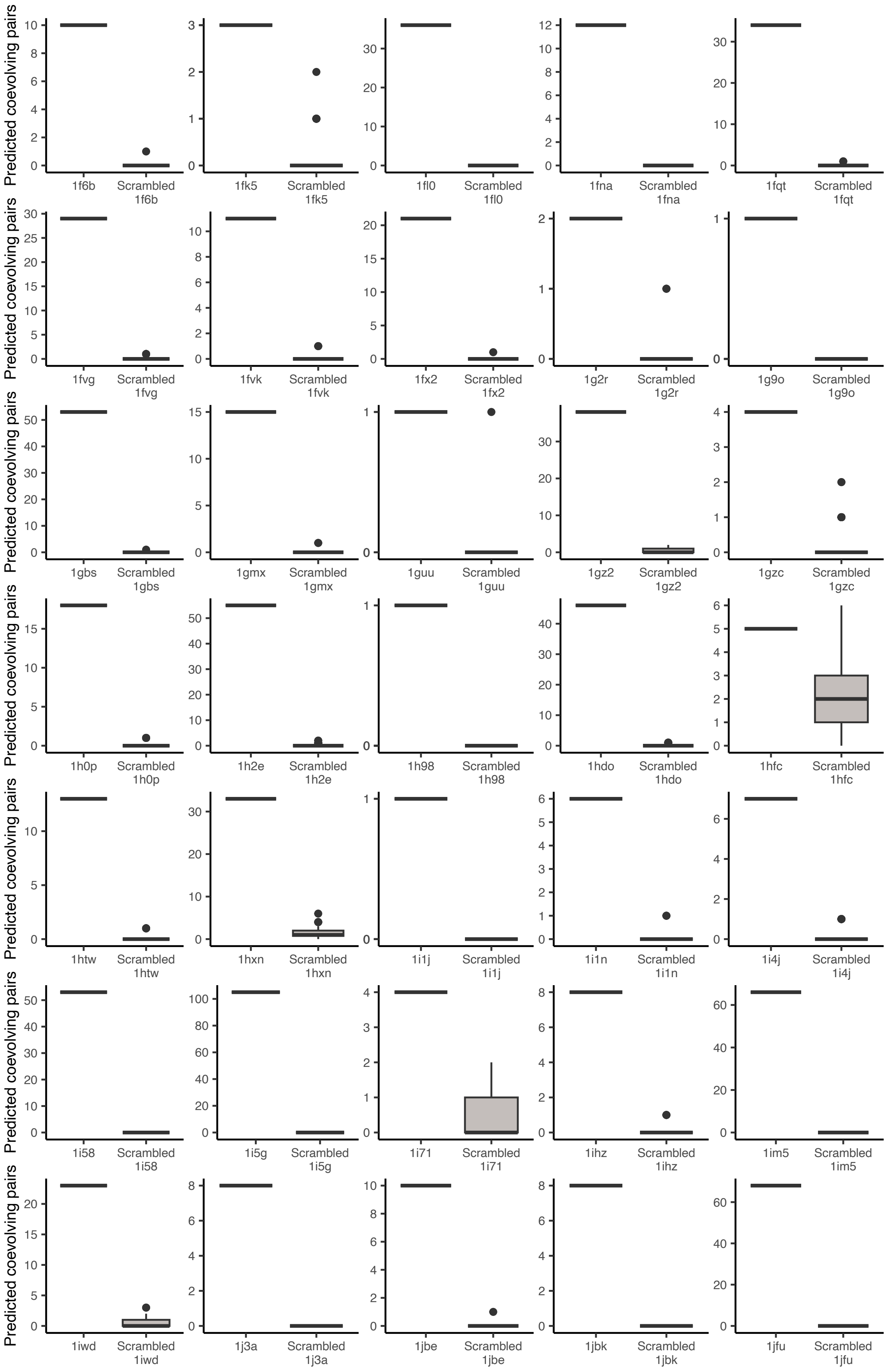
Predicted coevolving pairs
1f6b
Scrambled 1f6b
1fk5
Scrambled 1fk5
1fl0
Scrambled 1fl0
1fna
Scrambled 1fna
1fqt
Scrambled 1fqt
1fvg
Scrambled 1fvg
1fvk
Scrambled 1fvk
1fx2
Scrambled 1fx2
1g2r
Scrambled 1g2r
1g9o
Scrambled 1g9o
1gbs
Scrambled 1gbs
1gmx
Scrambled 1gmx
1guu
Scrambled 1guu
1gz2
Scrambled 1gz2
1gzc
Scrambled 1gzc
1h0p
Scrambled 1h0p
1h2e
Scrambled 1h2e
1h98
Scrambled 1h98
1hdo
Scrambled 1hdo
1hfc
Scrambled 1hfc
1htw
Scrambled 1htw
1hxn
Scrambled 1hxn
1i1j
Scrambled 1i1j
1i1n
Scrambled 1i1n
1i4j
Scrambled 1i4j
1i58
Scrambled 1i58
1i5g
Scrambled 1i5g
1i71
Scrambled 1i71
1ihz
Scrambled 1ihz
1im5
Scrambled 1im5
1iwd
Scrambled 1iwd
1j3a
Scrambled 1j3a
1jbe
Scrambled 1jbe
1jbk
Scrambled 1jbk
1jfu
Scrambled 1jfu

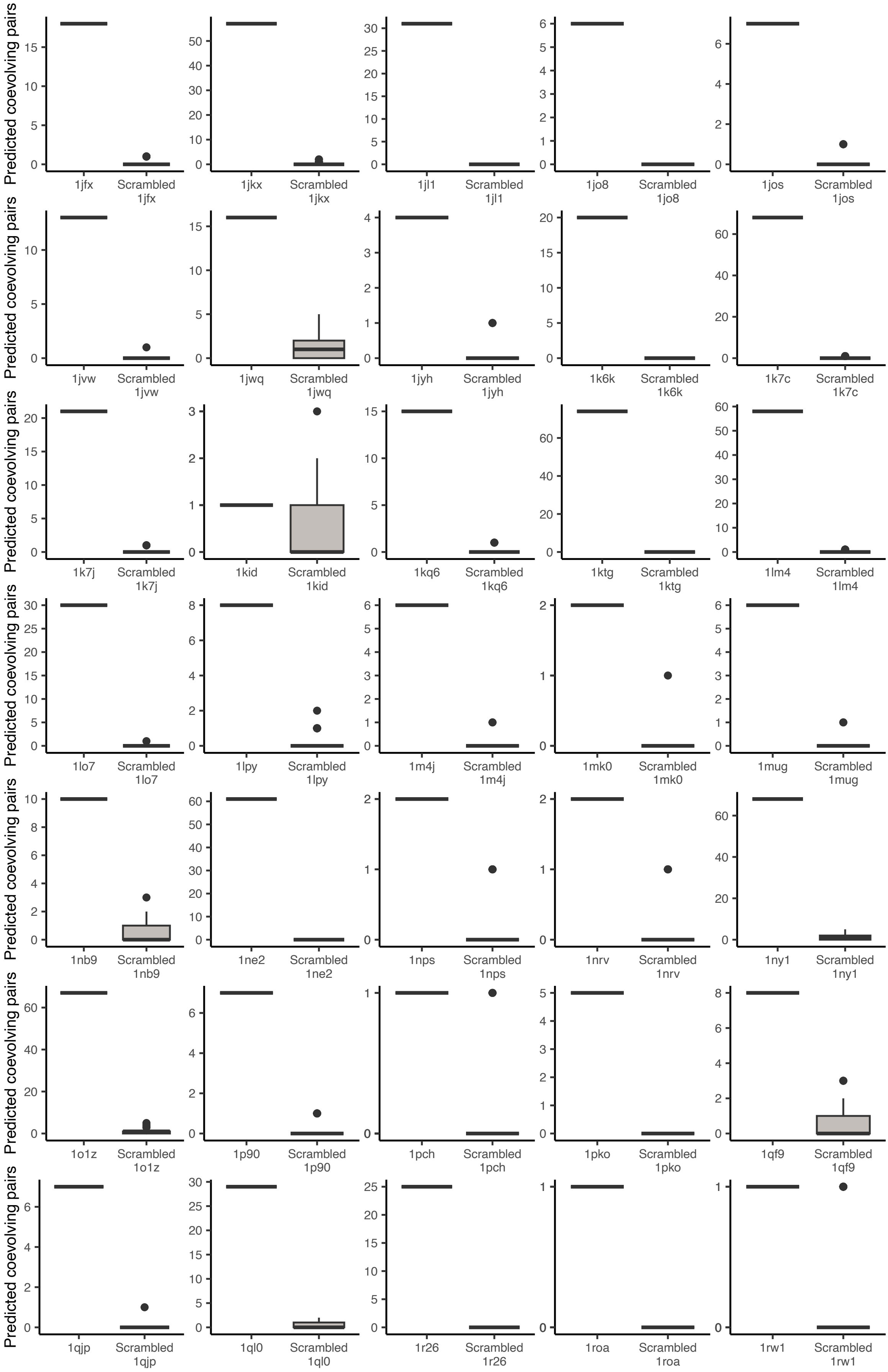
Predicted coevolving pairs
1jfx
Scrambled 1jfx
1jkx
Scrambled 1jkx
1jl1
Scrambled 1jl1
1jo8
Scrambled 1jo8
1jos
Scrambled 1jos
1jvw
Scrambled 1jvw
1jwq
Scrambled 1jwq
1jyh
Scrambled 1jyh
1k6k
Scrambled 1k6k
1k7c
Scrambled 1k7c
1k7j
Scrambled 1k7j
1kid
Scrambled 1kid
1kq6
Scrambled 1kq6
1ktg
Scrambled 1ktg
1lm4
Scrambled 1lm4
1lo7
Scrambled 1lo7
1lpy
Scrambled 1lpy
1m4j
Scrambled 1m4j
1mk0
Scrambled 1mk0
1mug
Scrambled 1mug
1nb9
Scrambled 1nb9
1ne2
Scrambled 1ne2
1nps
Scrambled 1nps
1nrv
Scrambled 1nrv
1ny1
Scrambled 1ny1
1o1z
Scrambled 1o1z
1p90
Scrambled 1p90
1pch
Scrambled 1pch
1pko
Scrambled 1pko
1qf9
Scrambled 1qf9
1qjp
Scrambled 1qjp
1ql0
Scrambled 1ql0
1r26
Scrambled 1r26
1roa
Scrambled 1roa
1rw1
Scrambled 1rw1

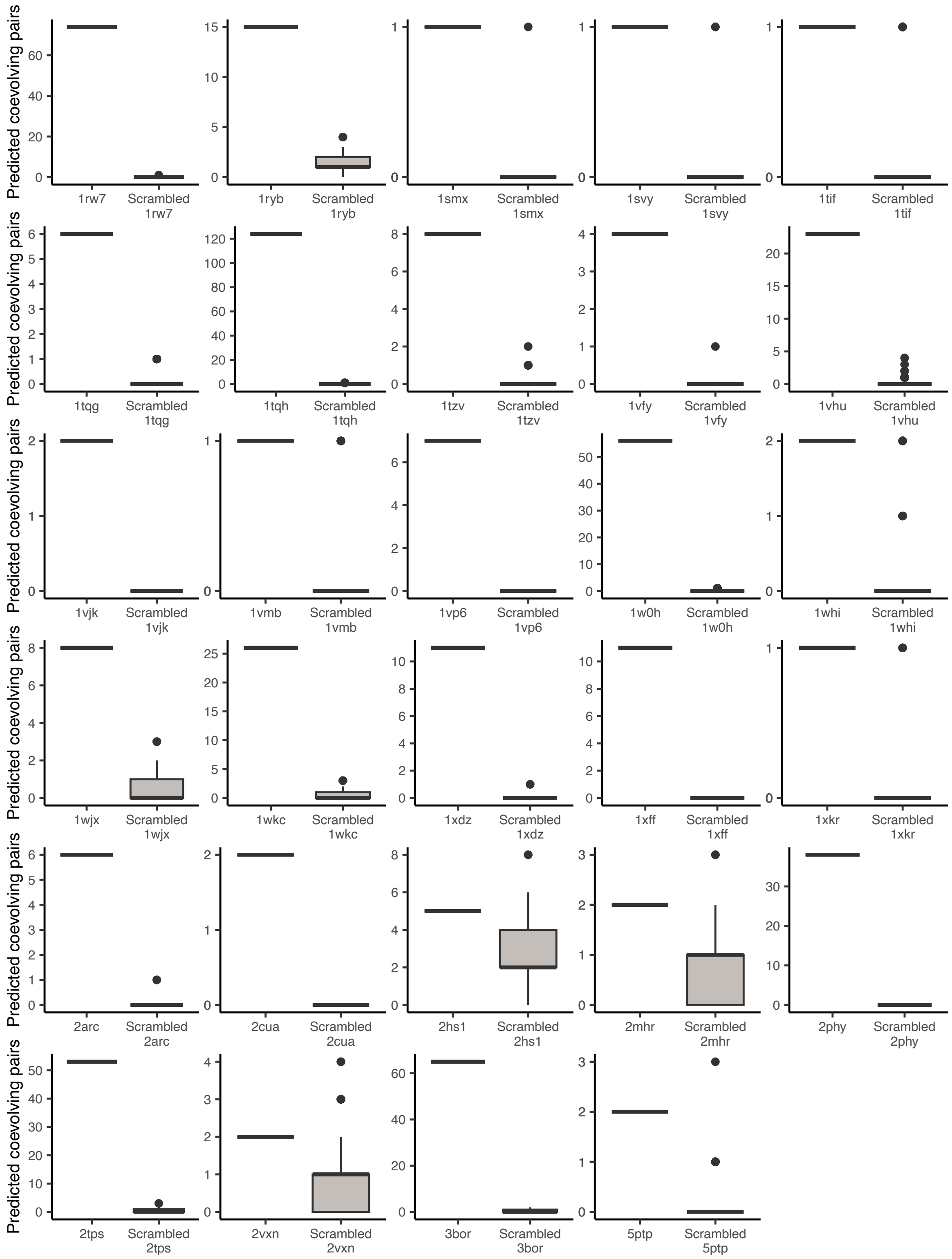

The number of predicted coevolving pairs predicted using the Box-Cox model and nodes method in each PSICOV sample versus the numbers predicted in 100 shuffled versions of the alignment.

Supplementary Figure 8. Comparisons of predicted coevolving pairs versus PSICOV and EVCouplings

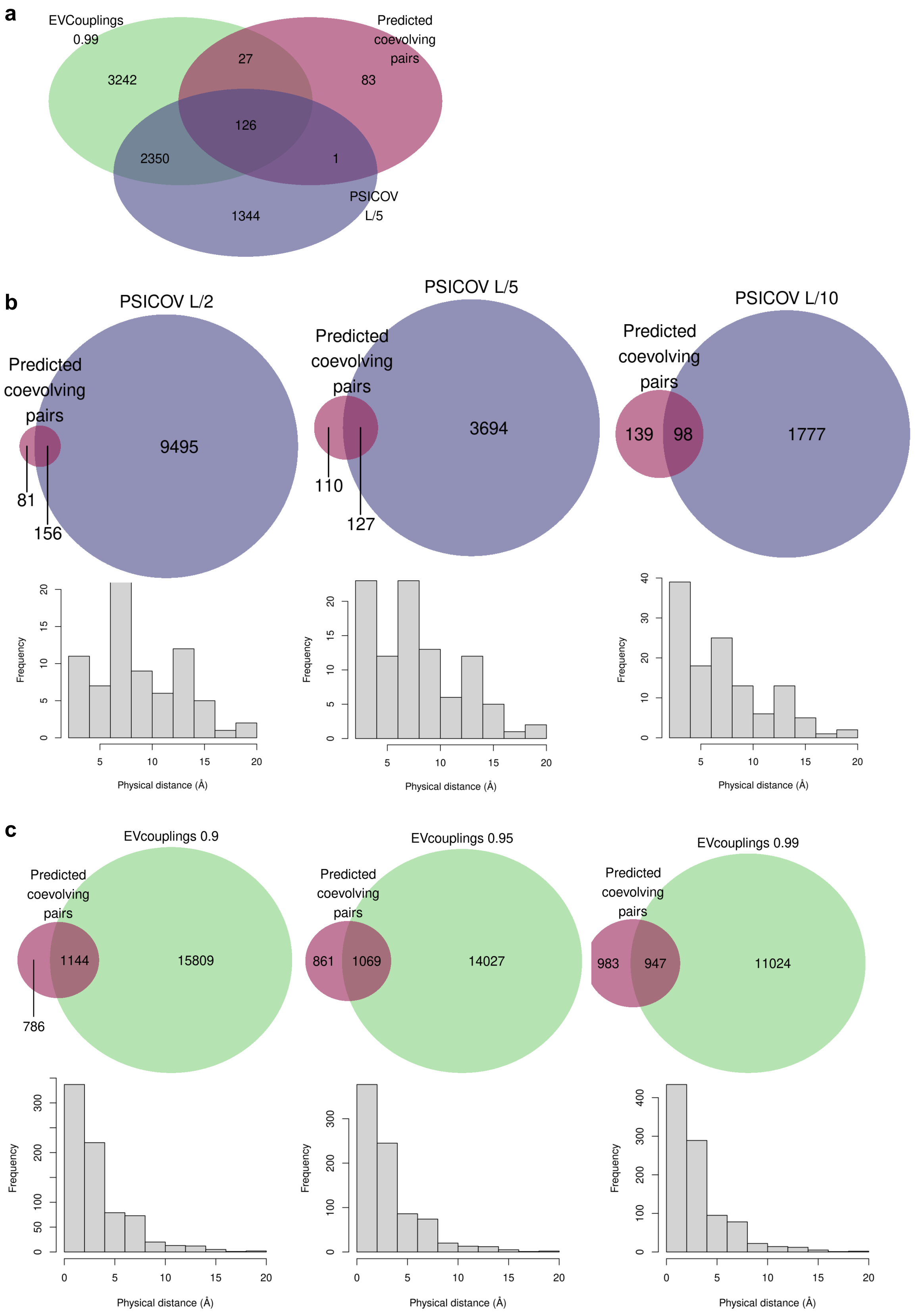

a, Overlap between pairs predicted to be coevolving by our method and predicted covarying pairs by PSICOV with L/5 cut-off and EVCouplings with 0.99 threshold. **b,** Overlap between our method's predicted coevolving pairs and pairs predicted to be physically close by PSICOV at three different levels of filter. As PSICOV only considers residues greater than 4aa apart in sequence space, the same filter was applied to our predictions. Underneath each Venn shows the physical distance between the pairs uniquely predicted to be coevolving by our method compared versus PSICOV at the given threshold. **c,** Overlap between our method's predicted coevolving pairs and pairs predicted to be covariant by EVcouplings at three different probability thresholds. Underneath each Venn shows the physical distance between the pairs uniquely predicted to be coevolving by our method compared versus EVcouplings at the given threshold.

Supplementary Figure 9. Features of networks of coevolving pairs in the PSICOV dataset

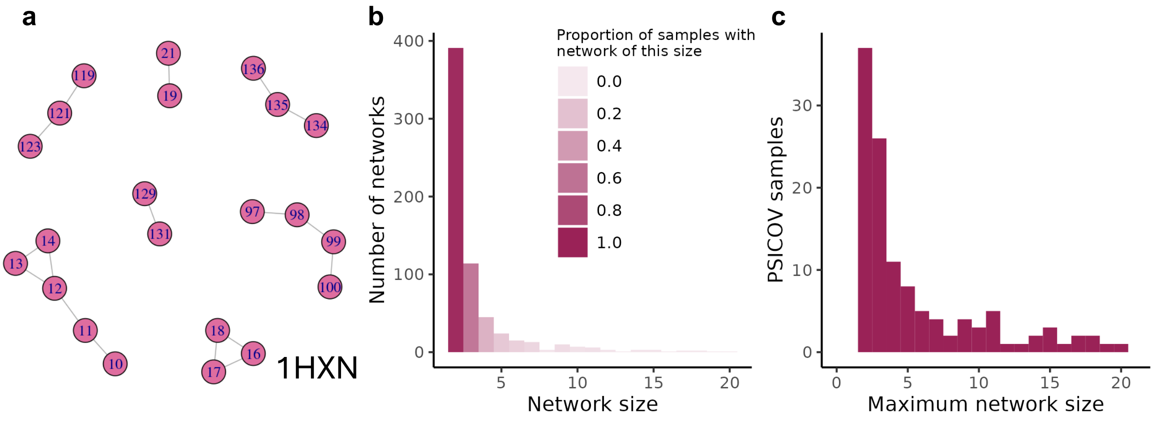

a, Coevolving pair predictions for the Hemopexin domain (represented by the PDB file 1HXN). Residues are depicted as nodes, labelled with their alignment position. Edges depict a coevolution relationship and illustrate how sets of pairs can form coevolution networks. The 17 coevolving pairs form two networks of two, three of three, one of four and one of five residues. **b,** Histogram of sizes of all networks formed by coevolving pairs across the PSICOV dataset. Bars are coloured by the proportion of samples that contained a network of that size. **c,** Histogram of the largest network formed by coevolving pairs for each sample in the PSICOV dataset.

Supplementary Figure 10. Features of predicted coevolving pairs in the PSICOV dataset

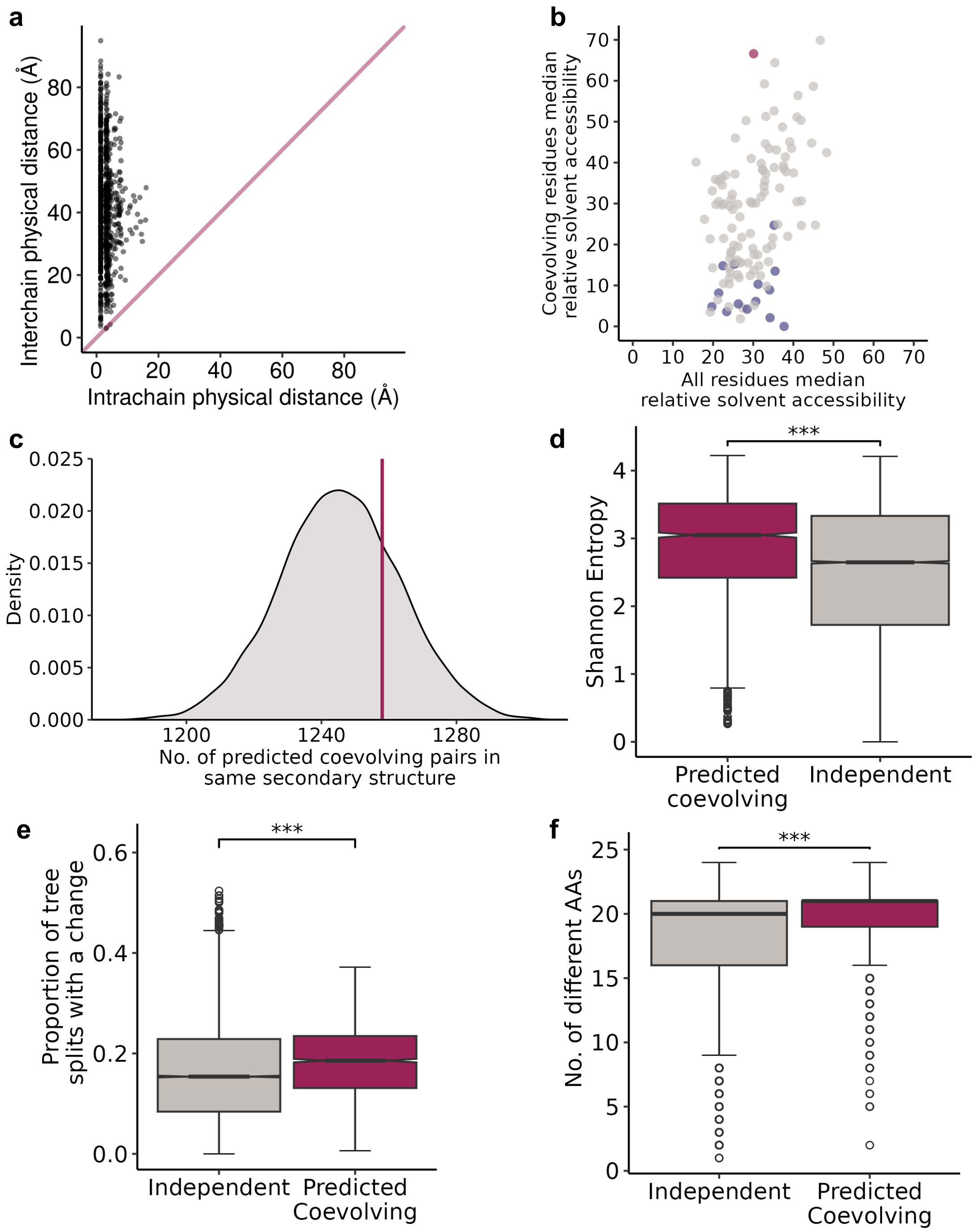

a, The physical distance between predicted coevolving pairs in the PSICOV dataset when inter-chain distances are considered. **b,** Median relative solvent accessibly of all residues versus those predicted to be coevolving in each PSICOV protein. Proteins with predicted coevolving residues statistically more surface or core than the overall protein are shown in red and blue respectively. **c,** Number of residues

predicted to be coevolving that reside within the same annotated piece of secondary structure. Shown against a background of random pairs selected with the same sequence space distance distribution. **d,** Shannon entropy of residues in predicted coevolving pairs versus all residues in the PSICOV dataset. **e,** Boxplot of the proportion of tree splits a position was observed to have changed in the tree reconstruction of residues predicted to be in a coevolving pair by both branches and nodes methods and independently evolving residues. Independently evolving residues are those not predicted to be coevolving by either the branches or nodes method. **f,** Boxplot of the number of different amino acids a position was observed to have changed in PSICOV alignments for residues predicted to be in a coevolving pair by both branches and nodes methods and independently evolving residues. Independently evolving residues are those not predicted to be coevolving by either the branches or nodes method. These alignments include gaps, selenocysteine (U) and ‘B’ (Asn or Asp), ‘X’ and ‘Z’ (Gln or Glu) ambiguity codes.

Supplementary Figure 11. Solvent accessibility coevolving pairs in samples with an observed statistical bias

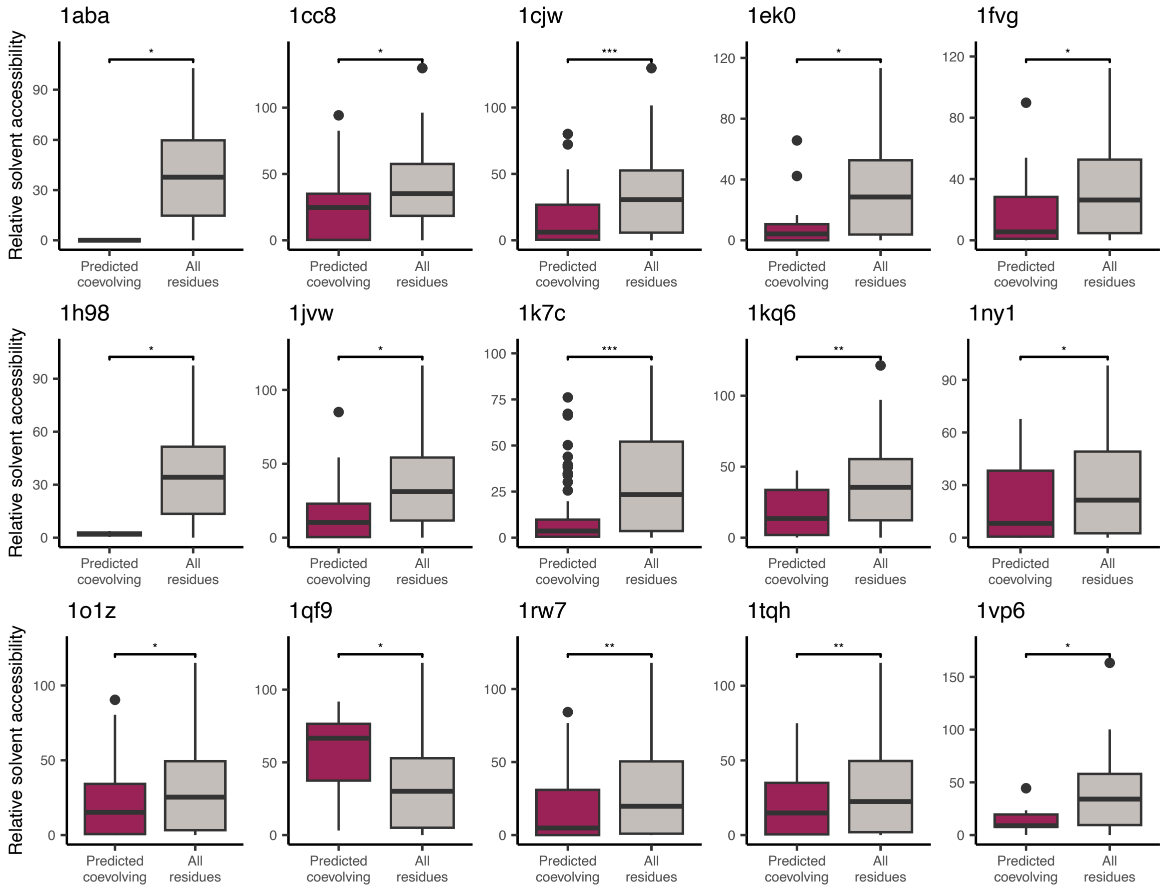

The relative solvent accessibility of residues predicted in coevolving pairs versus all pairs in the protein for each PSICOV sample with an observed statistical bias in Fig. S10b.

Supplementary Figure 12. Example amino acid favourability for two example coevolving pairs

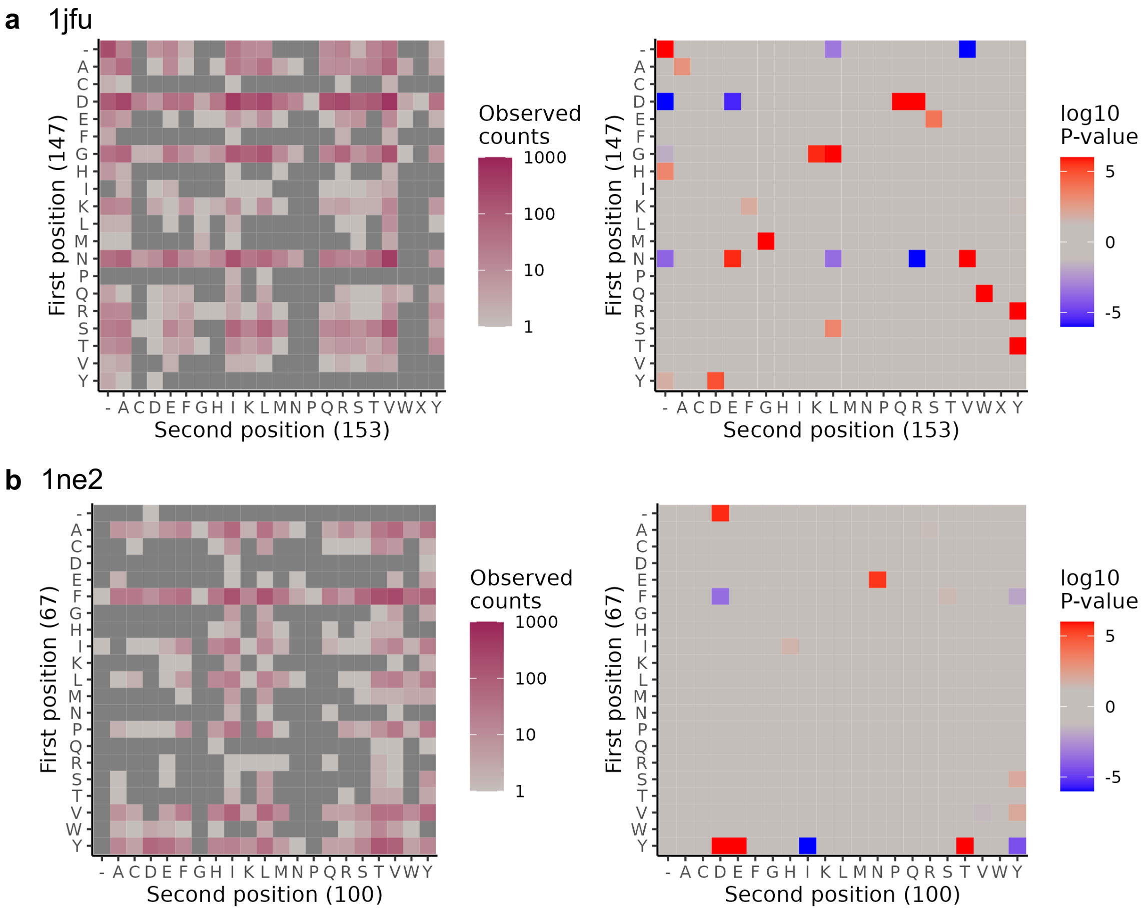

a, Heatmap of the counts of the ancestor states of amino acids in the reconstructed tree for alignment position 147 and 153 in the Redoxin domain (represented by PDB file 1JFU), as shown in Fig. 4a. Also shown is a heatmap illustrating the statistically significantly over- (red shades) and under- (blue shades) represented combinations. **b,** As **a,** but for residues 67 and 100 in the Ribosomal protein L11 methyltransferase (PrmA) domain (represented by PDB file 1NE2), illustrated in Fig. 4b.

Supplementary Figure 13. Impact of alignment features on numbers of predicted coevolving pairs

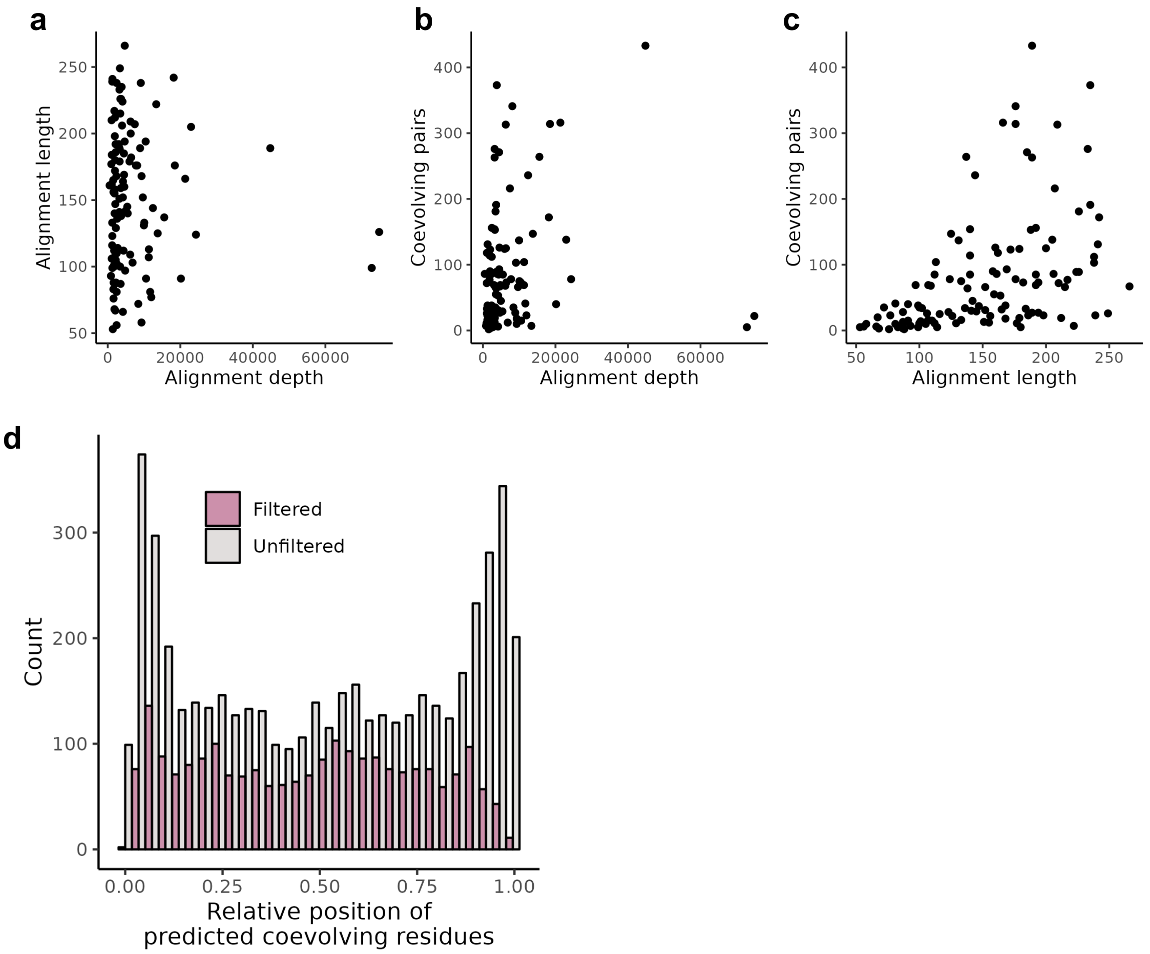

a, Length of PSICOV dataset sequences vs. the number of sequences in the alignment. **b,** Number of predicted coevolving pairs vs. the number of sequences in the alignment. **c,** Number of predicted coevolving pairs vs. length of PSICOV dataset sequences. **d,** Histogram of the relative position of residues predicted to be in a coevolving pair within an alignment before and after filtering for positions with more that 20% gaps.

Supplementary information about method development

for

Identification of Coevolving Positions by Ancestral Reconstruction

Table of Contents

Mapping amino acid substitutions to phylogenetic tree branches *27*

Ancestral reconstruction **27**

Backward & Forward traversing 27

Developing a method for the identification of co-evolution *30*

Rationale **30**

Data simulation and method characterisation *32*

Data Simulation **32**

Probability of sites being mutated 33

Conditional probabilities 33

Probability of mutations occurring during events 33

Performance metrics **33**

Precision 33

Specificity 33

Sensitivity 33

Combined measures 34

Methods for modelling S ~ D **34**

Linear modelling 34

Generalised linear modelling 35

Generalised additive modelling (GAM) 35

Prediction of coevolving pairs **36**

Linear modelling 36

Generalised linear modelling 36

Generalised additive modelling 37

Simulation 1: Independent evolution *37*

Simulation 2: 1 coevolving pair *40*

Simulation 3: 10 coevolving pairs *42*

Simulation 4: Multiple coevolving pairs – fixed matrix size *46*

Simulation 5: Multiple coevolving pairs – variable matrix size *50*

Branches and nodes overlap *52*

References *53*

Appendix: Parameters used in the simulations *54*

Mapping amino acid substitutions to phylogenetic tree branches

Ancestral reconstruction

Ancestral reconstruction is the process whereby the states of each node in the tree are inferred. Each column in the alignment is treated independently from the other columns.

Backward & Forward traversing

Traversing the tree from the leaves to the root, the algorithm identifies the nodes where a divergence occurred; i.e. the leaves or the child-nodes are not the same (see Fig. S14). Although some nodes are assigned a single state, others cannot be unambiguously resolved. A subsequent tree-transversion from the root to the leaves helps to increase the number of nodes with a single state. Knowing the states of the ancestor nodes and those of the children nodes, it is possible to infer in which branches of the tree the changes occur. All this information can be stored in a binary code (e.g. 0 for conservation, 1 for change), resulting in a big binary matrix containing information on the changes for each position in the MSA.

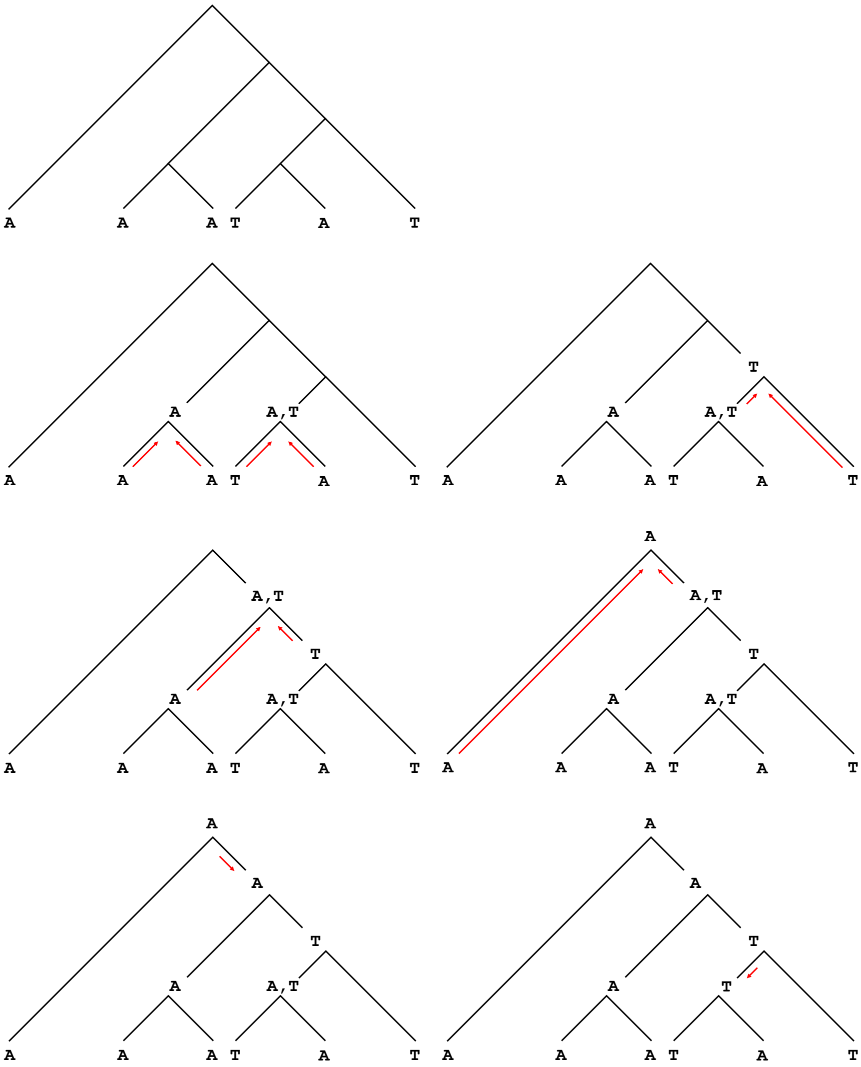

Supplementary Figure 14 Traversing the tree reveals most likely ancestral states and the branches at which changes have occurred (A and T represent observed residues at leaves and inferred ancestral states)

This information can be represented in a binary way at the nodes or branches level (see Fig. S15).

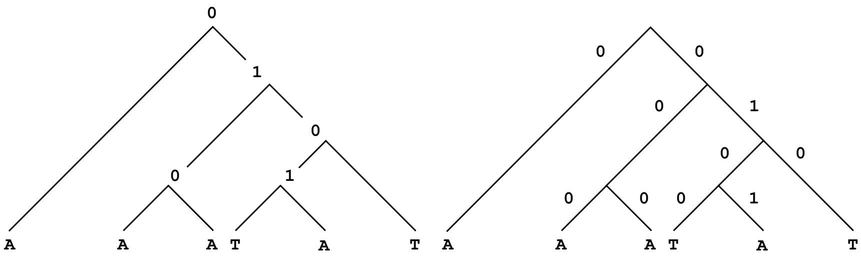

Supplementary Figure 15 Ancestral nodes and branches represented as binary change states (A and T represent observed residues at leaves)

Nodes cannot be fully solved in some instances. In those cases, we assigned a change state to both branches (see Figs. S16 and S17).

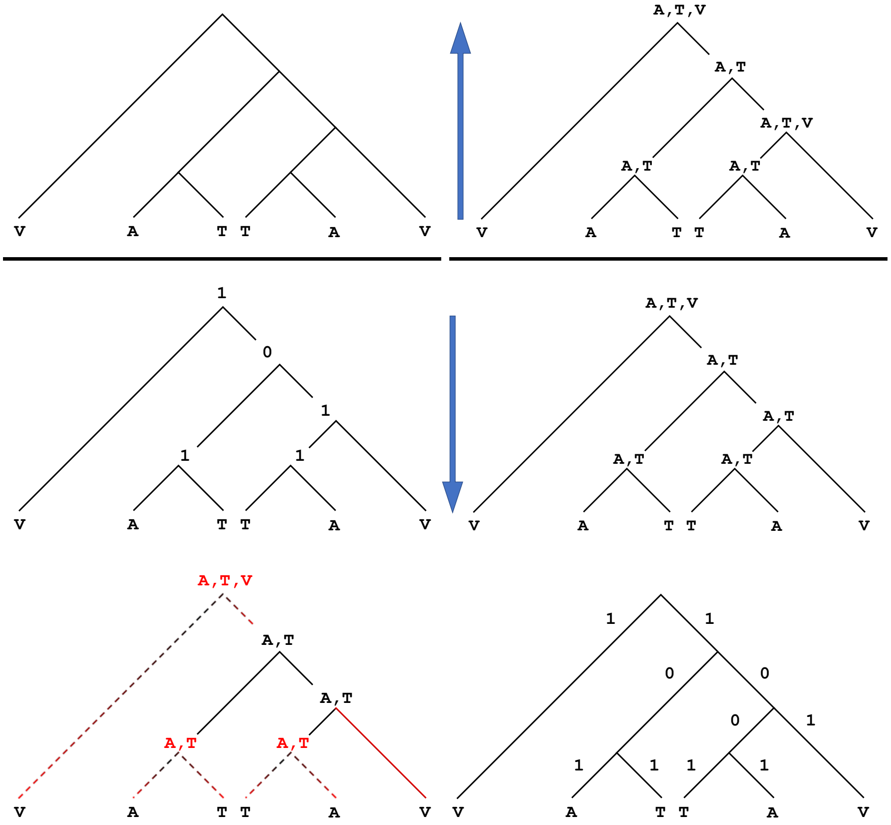

Supplementary Figure 16 Ancestral reconstruction with multiple instances of insufficient information for identifying the branches where the changes occurred (A, T and V represent observed residues at leaves and inferred ancestral states)

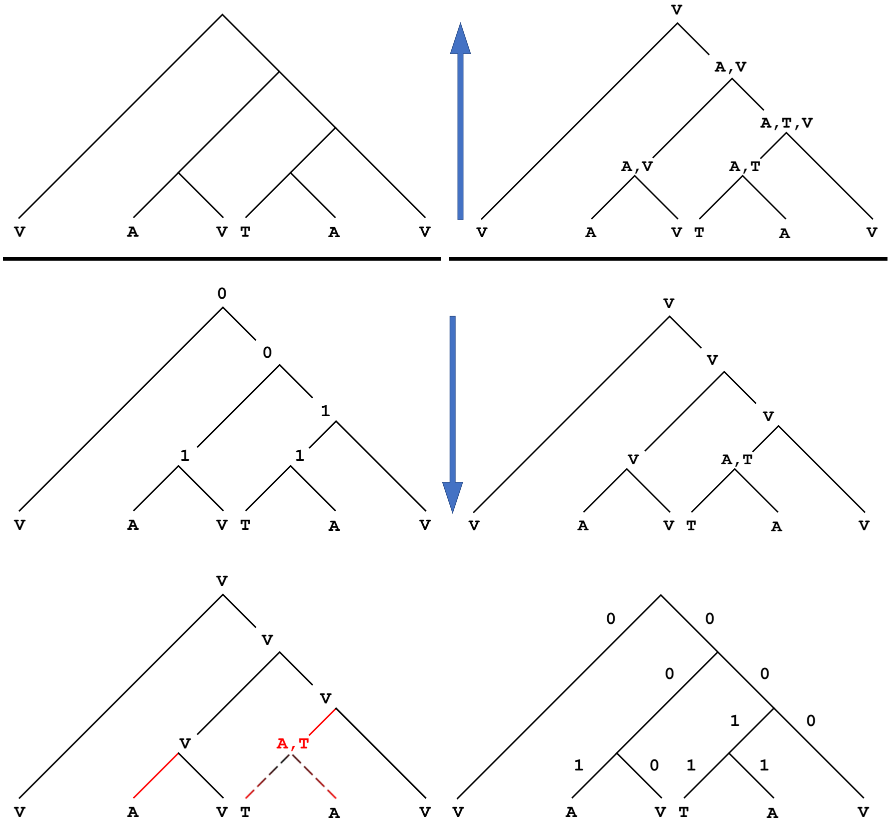

Supplementary Figure 17 Ancestral reconstruction with a single instance of insufficient information for identifying the branch where the change occurred (A, T and V represent observed residues at leaves and inferred ancestral states)

Developing a method for the identification of co-evolution

Rationale

Given two series of binary events, the probability of co-occurrence of particular outcomes depends on the frequency of each outcome in their respective series.

So, imagine that we have two series S_1 and S_2:

$$S_1 = \{0, 0, 0, 1, 0, 1, 0, 1, 0, 1\},$$

and

$$S_2 = \{1, 1, 1, 0, 0, 0, 0, 1, 1, 0\}.$$

The frequency of 1 in each of the series, $f_1(1)$ and $f_2(1)$, are 0.4 and 0.5 respectively. These also are the probability estimates of encountering a 1 in each of the series. Therefore, if we assume that these series are independent, the probability of a (1,1) co-occurrence is 0.2 (i.e. $P(1,1) = P_1(1) \times P_2(1)$). Given that probability and the number of trials (n = 10), the expected number or co-occurrences is 2; the 95% C.I. is (0,5).

If there are N series, the probability of pair-wise co-occurrence between series i and the rest of series will follow a linear trend whereby the higher the probability for the other series, the greater the probability of co-occurrence (see Supplementary Figure 18).

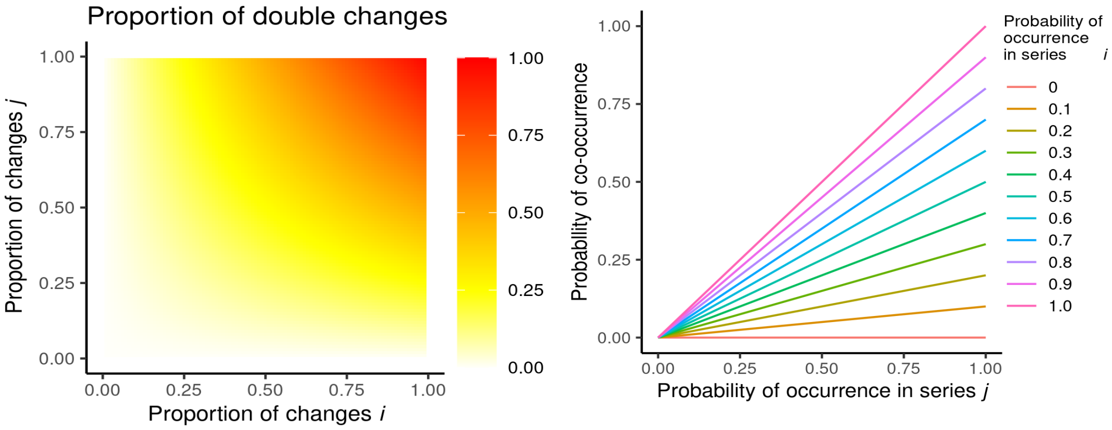

Supplementary Figure 18 Left, proportion of concurrent changes increases with proportion of changes in each series. Right, given a fixed probability of occurrence in series *i*, the probability of co-occurrence increases linearly with increasing probability in series *j*.

Conversely, the probability of single occurrences increases when one of the series has a high probability of successes while the probability is low in the other series (see Supplementary Figure 19). Again, the probabilities for N series will follow a linear trend; however, the slope of the line will be positive or negative depending on the probability in series i. If P_i is low, the higher the probability in the other series, the greater the probability for both separate and concurrent occurrences. If P_i is high, as the probability of co-occurrences increases, the probability of separate occurrences decreases.

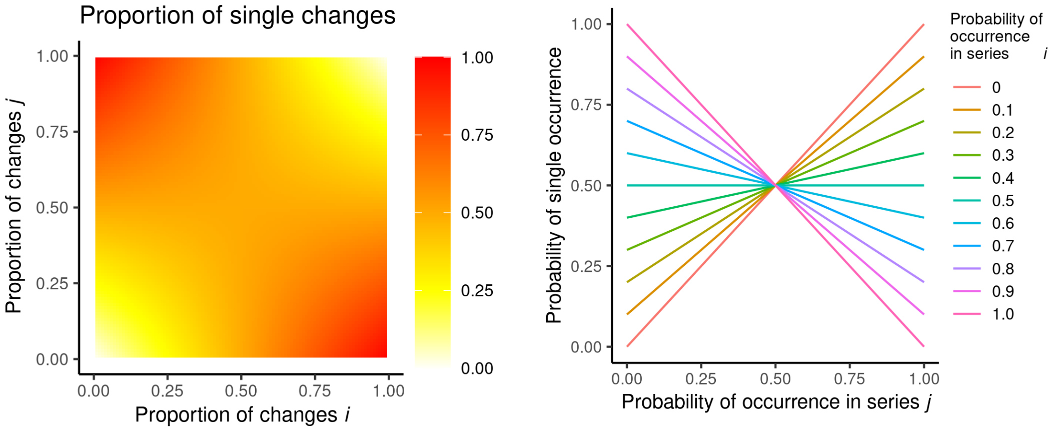

Supplementary Figure 19 Left, proportion of separate changes increases with diverging proportion of changes in each series. Right, given a fixed probability of occurrence in series *i*, the probability of separate occurrence increases or decreases linearly and inversely to changing probability in series *j*.

Given that there is an expected proportion of separate and concurrent occurrences for each combination of per-series probabilities of success, we can identify the area whereby the different combinations of co-occurrences and single occurrences will sit in a plot (see Supplementary Figure 20). This area is delimited by the Y-axis in the left (i.e. no co-occurrences at all), the $S = 1 - D$ line as the maximum proportion of single occurrences for a given proportion of co-occurrences, and a curve describing the expected minimum proportion of single occurrences if the series are independent. Thus, the proportion of single occurrences will only be lower than that minimum if the two series are co-dependent. Again, there are linear relationships for those variables for a given P_i: $P_S = \beta_0 + \beta_1 \boldsymbol{P_D}$'. As seen from this equation and Supplementary Figure 20, if P_i is above 0.5, P_D will increase with P_j, but P_S will decrease; so, β_1 will have a negative value. We only expect this to occur in cases of very accelerated evolution; e.g. in some virus.

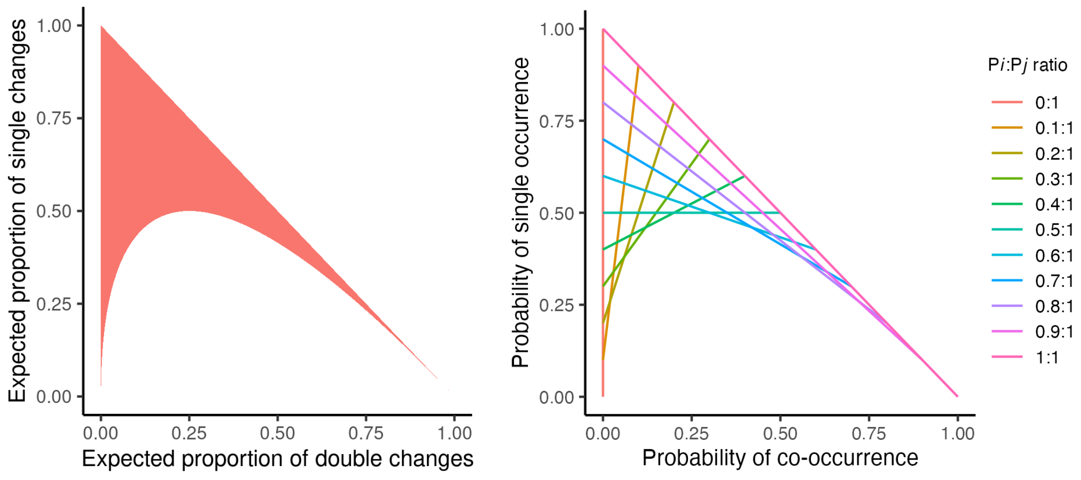

Supplementary Figure 20 Left, shadow shows the region where the expected proportions of concurrent and separate changes sit. Right, linear trend showing the change in probability of co-occurrence vs probability of single occurrence, given a fixed P*i*:P*j* ratio.

Data simulation and method characterisation

Data Simulation

An *N* number of independent evolutionary branching events were simulated by drawing a sample of random positions (i.e. the positions to be mutated). Each position had a different probability of being mutated. That probability was the same for all branching events. The number of positions to be mutated was variable between branching events.

Finally, probability of mutation for some positions was modified within a particular event depending on other positions being mutated.

Probability of sites being mutated

Each site was assigned a different probability of being changed during divergence events. The probabilities were drawn from a beta distribution. Probabilities are scaled so their sum equals 1.

Conditional probabilities

If two residues covary and one of them is changed, probability of being changed for the other residues is increased, and all the other probabilities are automatically rescaled. We used a multiplicative factor in order to model the increment of probability for covarying residues.

Probability of mutations occurring during events

Each divergence event was assigned a different probability to contain changes. The probabilities were drawn from a beta distribution. The number of changes occurring at a given event equal the rounded value of the probability times the number of sites.

The different simulation-specific probabilities (plus other parameters) are more thoroughly described when explaining the different simulation scenarios and summarised in an appendix at the end of this document.

Performance metrics

Precision

Precision measures the percentage of correct predictions over the number of positive predictions made. This is our metric of choice since we want to maximise the probability that our predictions are true positives. It can also be interpreted as 1 – FDR (False Discovery Rate); so, the higher the precision, the lower the FDR.

Specificity

Specificity measures the percentage of correct predictions over the number of negative cases. Thus, it measures the ability to not misclassify as positives the true negatives.

Sensitivity

Sensitivity (also known as recall) is not a good metric in our case. The reason is that previous research demonstrated that the number of detectable covarying pairs that we should expect in a MSA is small (Talavera, Lovell et al. 2015); probably, much smaller than the actual number of covarying pairs. Therefore, we should expect low sensitivity

figures. If we tried to maximise the sensitivity hence increasing the number of true positives that we identified, we would likely increase the number of false positives as well.

Combined measures

Markedness and informedness are two complementary metrics that try to assess the quality of the method while correcting for the biasing effect of prevalence in some of the simplest metrics (Powers 2011).

Markedness quantifies the probability that the true nature of the pair (either covarying or varying independently) is marked by the prediction; i.e. which percentage of positive and negative predictions are true positives and true negatives, respectively. It is calculated as the sum of the positive and negative predictive values minus 1. The positive predictive value is an alternative name for precision.

Informedness represents the probability of an informed decision (vs a guess). It is calculated as the sum of sensitivity and specificity minus 1. Since we are trying to maximise the specificity of the method, while not considering its sensitivity, it is very likely that informedness would be low in most instances.

Methods for modelling S ~ D

We used linear modelling, generalised linear modelling and generalised additive modelling in order to model the S ~ D dependency.

Linear modelling

Linear modelling predicts that constant changes in the independent variable (i.e. *D*) will result in constant changes in the response variable (i.e. *S*), hence it is a linear response model. We used 3 different modelling approaches:

Linear regression

$$S_i = \beta_0 + \beta_1 D_i + \varepsilon_i$$

Linear regression of the logarithm-transformed dependent variable

$$\ln(1 + S_i) = \beta_0 + \beta_1 D_i + \varepsilon_i$$

One count is added to the response variable in order to avoid ln(0).

Box-Cox transformation of the dependent variable followed by linear regression

The response variable (S_i) is transformed based on the optimal λ value ($S'_{\lambda,i}$). λ is estimated by fitting the $S'_{\lambda,i} = \beta_0 + \beta_1 D_i + \varepsilon_i$ model, and selecting the λ that maximises the log-likelihood profile.

We used two alternative forms of the transformation. For cases where min(S) > 0, we used the original transformation:

$$S'_{\lambda,i} = \begin{cases} \dfrac{(S_i)^\lambda - 1}{\lambda} & \text{if } |\lambda| > 10^{-6} \\ \ln(S_i) & \text{if } |\lambda| \le 10^{-6} \end{cases}$$

While we used the Box-Cox transformation with negatives for cases where min(S) = 0:

$$S'_{\lambda,i} = \begin{cases} \dfrac{\left(0.5 \times (S_i + s_i)\right)^\lambda - 1}{\lambda} & \text{if } |\lambda| > 10^{-10} \\ \ln\left(0.5 \times (S_i + s_i)\right) & \text{if } |\lambda| \le 10^{-10} \end{cases}$$

where $s_i = \sqrt{S_i^2 + \gamma^2}$. Since we used $\gamma = 10^{-9}$, $s_i \approx S_i$ hence both families of transformations return very similar results, but the latter avoids ln(0) when λ is very close to 0.

Generalised linear modelling

Generalised linear models assume that the response variable does not vary linearly with the independent variable, but that changes in the response variable follow an exponential distribution.

Generalised linear models have 3 components:

- An exponential distribution for the variation of the response variable
  We used 2 different distributions:
  - Poisson distribution
  - Negative Binomial distribution
- A linear predictor: $\eta = \boldsymbol{D}\beta$
- A link function g such that $E(S|D) = \mu = g^{-1}(\eta)$
  We used the logarithm function as the link function in both cases:
  $\boldsymbol{D}\beta = \ln(\mu)$

Generalised linear models were fitted by estimating the unknown parameters β using an iteratively reweighted least squares algorithm.

Generalised additive modelling (GAM)

Generalised additive models are an extension of generalised linear models, whereas variation in the response variable depends on the linear combination of some smooth functions:

$$g\big(E(S)\big) = \beta_0 + f_1(D_1) + f_2(D_2) + \cdots + f_m(D_m)$$

Variation of the response variable is assumed to follow an exponential distribution. We used two such distributions:

- Poisson distribution
- Negative Binomial distribution

The logarithm function was used as the link function in both cases.

Prediction of coevolving pairs

After modelling the S ~ D dependency, we estimated which range of S values were expected for each D value. Coevolving pairs are instances whereby the number of separate changes is smaller than the one expected, given the number of concurrent changes observed between the positions. Summing up, the occurrences of separate and concurrent changes do not follow the model of independent evolution, and they are enriched in co-occurrences (i.e. concurrent changes).

Linear modelling

The prediction interval was calculated with the *predict()* function within the *stats* R package. This generic function invokes the method corresponding to the fitted model; i.e. *predict.lm()*. This method permits predicting two types of intervals: the confidence intervals, and the prediction intervals. The confidence interval represents the uncertainty around the expected value of the dependent variable given an observation of the independent variable; i.e. uncertainty around the mean response. The prediction interval represents the uncertainty around a single value; i.e. the whole range of possible values for the dependent variable given that observation.

Generalised linear modelling

The *predict.glm()* method does not permit calculating prediction intervals. Therefore, we had to use some empirically-built prediction intervals. We used the bootstrapping approach presented by WenSui Lui (Lui 2015).

```
GENERATE n seed numbers
FOREACH seed number
    SAMPLE with replacement from the model data
    Update the model with sampled data
    Predict response values for pdata from the updated model
    Generate a Poisson distribution with lambda as the response
    values and randomly draw from the distribution for each pdata

CALCULATE quantile values to satisfy p for each point in pdata
```

Where *model* is the generalised linear model fitted with the *glm()* function, *pdata* is the range of points we want to predict the intervals for, *n* is the number of bootstraps used (1,000 in this case), and *p* is the level parameter (i.e. how wide the range of predictions should be). Finally, the lower and upper limits of the prediction interval for each predicted point are returned.

Briefly, the model data is bootstrapped by taking a sample with replacement of the same size. That bootstrapped data is used to update the model, and response values are predicted for each value within *pdata*. Those response values are used as the lambda values for randomly drawing numbers from a Poisson distribution, one number for each *pdata* value. After repeating the process *n* times, the quantile values that satisfy the *p* level are calculated for each value of *pdata*.

Generalised additive modelling

The predicted values and their standard errors were predicted using the *predict()* function. Those values were transformed to the correct range of values by using the inverse of the link function. The quantile functions for the Poisson and Negative Binomial distributions – respectively *qpois()* and *qnbinom()* within the *stats* R package- were used to calculate the prediction intervals depending on which distribution was used in the modelling.

Simulation 1: Independent evolution

Each tree-split event is treated as independent. For each split, a number of positions are randomly selected as having changed at either the right or the left branch of the tree, or in both branches.

We used different beta distributions in order to simulate different evolutionary rates for each position (i.e. different probability of being replaced), see Supplementary Figure 21. Left and right branches have the same probability of being changed.

Beta(1,5) median 0.129, Beta(1,10) median 0.067, Beta(2,10) median 0.148, Beta(2,20) median 0.079, Beta(3,15) median 0.154, Beta(3,30) median 0.083, Beta(4,20) median 0.157, Beta(4,40) median 0.085, Beta(5,25) median 0.159, Beta(5,50) median 0.086.

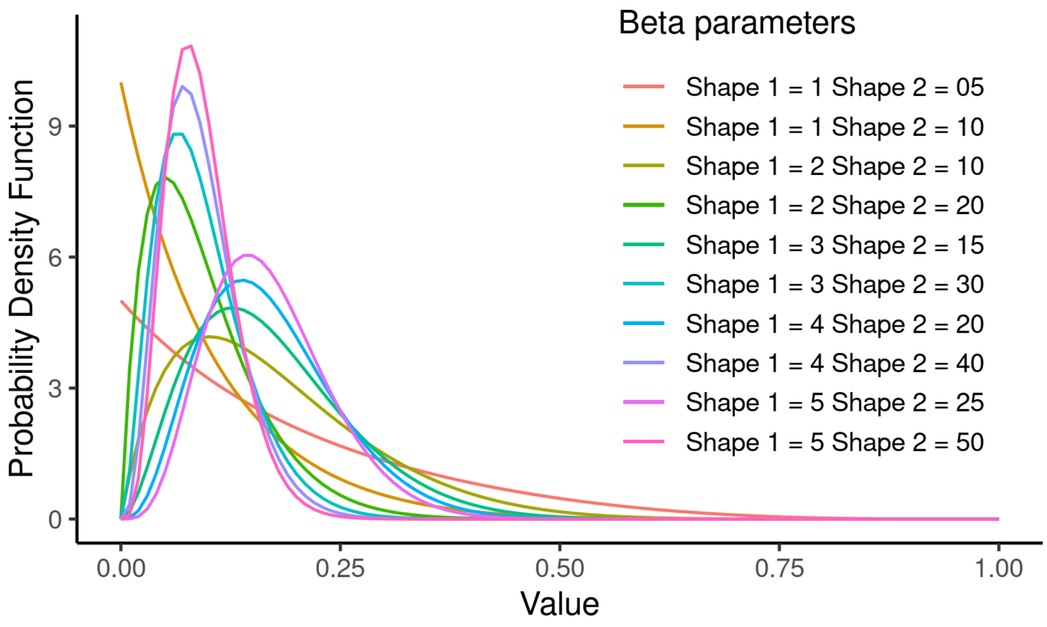

Supplementary Figure 21 Probability density plots of Beta values used to determine probability of positions being replaced and the proportion of positions replaced in each tree bifurcation.

We used the same distributions in order to assign the proportion of positions that needed to be replaced in each tree bifurcation. Thus, the number of replacements within the *M x N* matrix is *N x Beta*(α,β), where N is the number of positions. The number of replacements per split/branch can be seen as a very rough representation of different branch lengths. Each combination of evolutionary rate and proportion of position beta distributions (100 combinations) was simulated 100 times for a total of 10,000 simulations.

Supplementary Table 1 shows that the GLM models and the GAM using a Poisson distribution to model the response variable predict many positives even if there are none. Therefore, we decided not to use those models in subsequent analyses. Moreover, the table also shows that there are no big specificity differences when analysing the splits at the nodes or branches level.

Supplementary Table 1 Performance of different combinations of models and methods for identifying coevolving positions in an independent-evolution scenario

Model	Method	Mean FP per simulation	Median FP per simulation
Linear model	Branches	0.142	0
Logarithmic model	Branches	0.060	0
Box-Cox Transform model	Branches	0.013	0
Poisson Regression model	Branches	62.930	36
Negative binomial regression model	Branches	62.143	36
Poisson GAM	Branches	10.267	2
Negative binomial GAM	Branches	1.948	0
Linear model	Nodes	0.075	0
Logarithmic model	Nodes	0.066	0
Box-Cox Transform model	Nodes	0.015	0
Poisson Regression model	Nodes	48.142	25
Negative binomial regression model	Nodes	47.572	25
Poisson GAM	Nodes	6.509	1
Negative binomial GAM	Nodes	1.077	0

In addition, we examined the distance distribution of the false positive predicted coevolving pairs. Supplementary Figure 22 shows the distribution of distances between false positive predictions using the branches approach, versus a distribution of 100,000 randomly selected pairs between one and 100. Differences in the distributions are minor and explained by the low total numbers of false positive predictions in the dataset e.g. only 127 for Box-Cox.

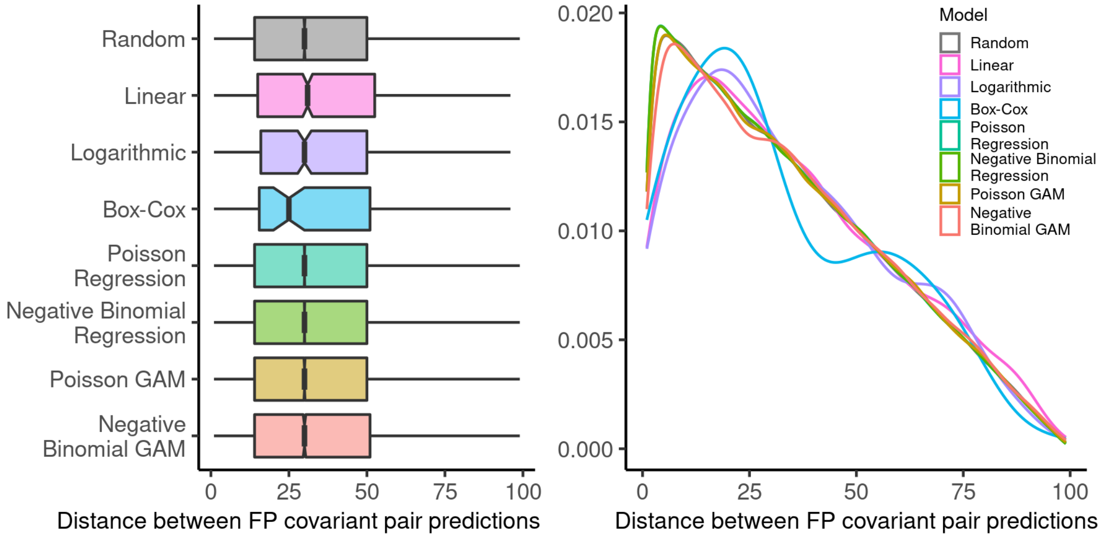

Supplementary Figure 22 Box plot and density function of the distance between false positive covariant pair predictions for each model using the branches method. A distribution for 100,000 randomly selected pairs is also shown

Simulation 2: 1 coevolving pair

We used the same Beta distributions in order to model the position-specific evolutionary rates, and the different branch lengths (i.e. the number of replacements per tree bifurcation).

Two positions were co-dependent; i.e. if one of those two positions was randomly selected to be replaced in a particular split event (in a specific branch), the other position's probability of being selected had a X-fold increase for that event (in the same branches), see Supplementary Table 2. The probabilities for the rest of positions were not modified. Coevolution only had effect on one particular branch, and it did not increase the probability of replacement for the equivalent co-dependant in the other branch. Since the sum of all probabilities are scaled to 1, the probability for the co-dependent pair was increased while the rest were slightly decreased. This only affected that particular event; i.e. each event started with the original Beta-distributed probabilities.

Supplementary Table 2 Multiplicative factors for the different simulation examples

Example	Position 1	Position 2	Covariation multiplicative factor
1	35	90	2
2	35	90	3
3	35	90	4
4	35	90	5
5	35	90	7
6	35	90	9
7	35	90	11
8	35	90	14
9	35	90	17
10	35	90	20

Each combination of evolutionary rate and proportion of position beta distributions and covariation factor (1,000 combinations) was simulated 100 times for a total of 100,000 simulations.

Supplementary Table 3 show how the linear models are much better at predicting the true positives than the GAM using a Negative Binomial distribution. Therefore, we selected them for further analyses. Again, no big performance differences are observed when analysing the splits at the branches or nodes level.

Supplementary Table 3 Performance of different combinations of models and methods for identifying coevolving positions in a single coevolving-pair scenario

Model	Method	Precision	Specificity	Markedness	Informedness
Linear	Branches	0.633	0.999972	0.633	0.243
Logarithmic	Branches	0.812	0.999988	0.812	0.247
Box-Cox	Branches	0.945	0.999997	0.945	0.236
Negative binomial GAM	Branches	0.003	0.999602	0.002	0.005
Linear	Nodes	0.743	0.999984	0.743	0.220
Logarithmic	Nodes	0.793	0.999987	0.792	0.233
Box-Cox	Nodes	0.932	0.999997	0.932	0.202
Negative binomial GAM	Nodes	0.002	0.999780	0.002	0.002

Supplementary Figure 23 shows the Recall of the linear models and how it varies with the increase of the covariation multiplicative factor. To achieve recall of above 0.25 a covariation factor greater than seven is required.

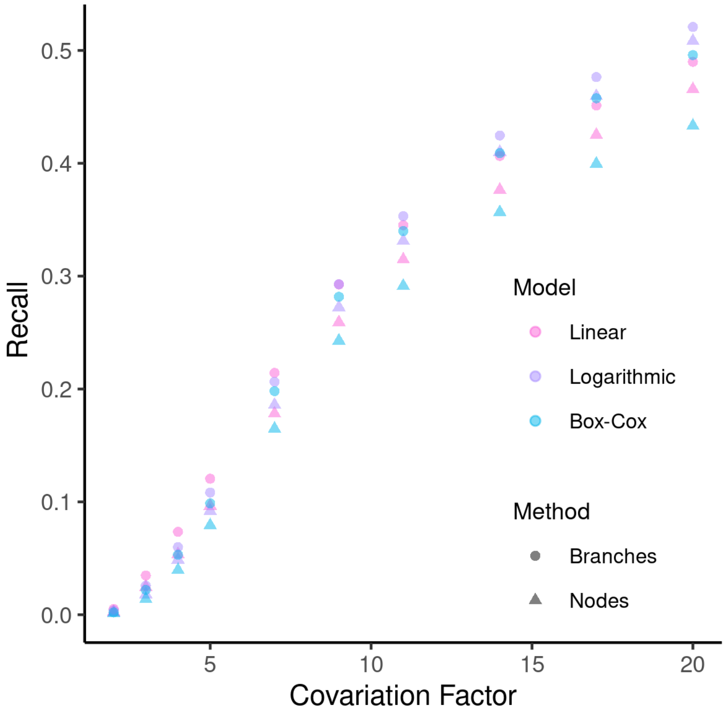

Supplementary Figure 23 Recall values for the linear models using both the branches and nodes methods at each level of covariation factor.

Simulation 3: 10 coevolving pairs

Simulations were run as previously stated. The only difference is that each simulation contained 10 coevolving pairs instead of just one. Those pairs had been randomly selected, and represented a mixture of close-, mid- and long-range interactions (Supplementary Figure 24).

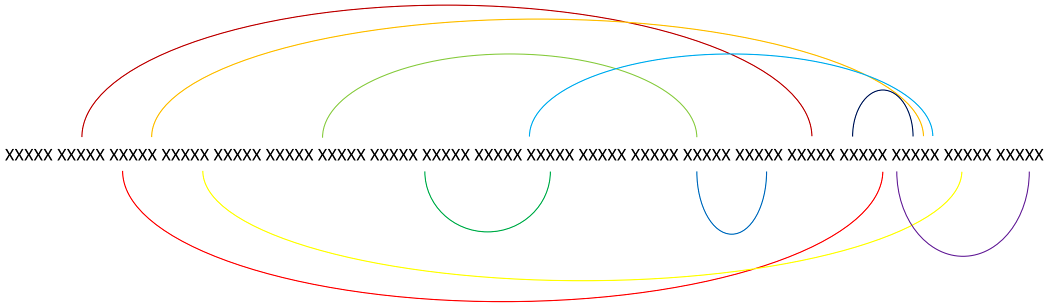

Supplementary Figure 24 Distribution of the 10 coevolving pairs. Curved lines show which positions are linked.

The multiplicative values were randomly drawn from Normal distributions with different mean and standard deviation for each model (Supplementary Table 4). The values were rounded to the closest integer.

Supplementary Table 4 Distribution of multiplicative factors for the different simulation examples

Example	Distribution	Multiplicative factors
1	$\mathcal{N}(4,1)$	3, 3, 3, 3, 4, 4, 5, 5, 5, 5
2	$\mathcal{N}(5,1)$	4, 5, 5, 5, 5, 6, 6, 6, 6, 6
3	$\mathcal{N}(6,1)$	4, 5, 5, 6, 6, 6, 6, 6, 6, 7
4	$\mathcal{N}(8,2)$	5, 7, 8, 9, 9, 9, 10, 10, 11, 13
5	$\mathcal{N}(10,2)$	7, 7, 7, 8, 9, 9, 10, 10, 10, 11
6	$\mathcal{N}(12,2)$	10, 11, 11, 11, 11, 12, 12, 14, 14, 15
7	$\mathcal{N}(15,3)$	12, 12, 13, 13, 14, 15, 15, 16, 19, 21
8	$\mathcal{N}(18,3)$	14, 15, 20, 20, 21, 22, 22, 23, 24, 24
9	$\mathcal{N}(21,3)$	18, 18, 20, 20, 21, 22, 23, 24, 25, 26
10	$\mathcal{N}(25,4)$	22, 22, 23, 24, 25, 25, 26, 27, 28, 29

Each combination of evolutionary rate and proportion of position beta distributions and covariation factor distribution (1,000 combinations) was simulated 100 times for a total of 100,000 simulations.
Results shown in Supplementary Table 5 suggest that although all models are extremely precise and specific, the Box-Cox transform might be the best one.

Supplementary Table 5 Performance of different combinations of models and methods for identifying coevolving positions in a multiple coevolving-pair scenario

Model	Method	Precision	Specificity	Markedness	Informedness
Linear	Branches	0.962	0.999986	0.961	0.316
Logarithmic	Branches	0.984	0.999989	0.983	0.325
Box-Cox	Branches	0.996	0.999998	0.995	0.314
Linear	Nodes	0.977	0.999975	0.976	0.289
Logarithmic	Nodes	0.982	0.999990	0.981	0.309
Box-Cox	Nodes	0.996	0.999997	0.994	0.279

Looking at the effect of different covariation factors it appears that below certain levels of coevolution, detection becomes much less likely (see Fig. S25 for Box-Cox results). This

agrees with the single coevolving pair simulations that suggested a multiplicative value of greater than seven would be required to achieve recall of greater than 25% (Fig. S23).

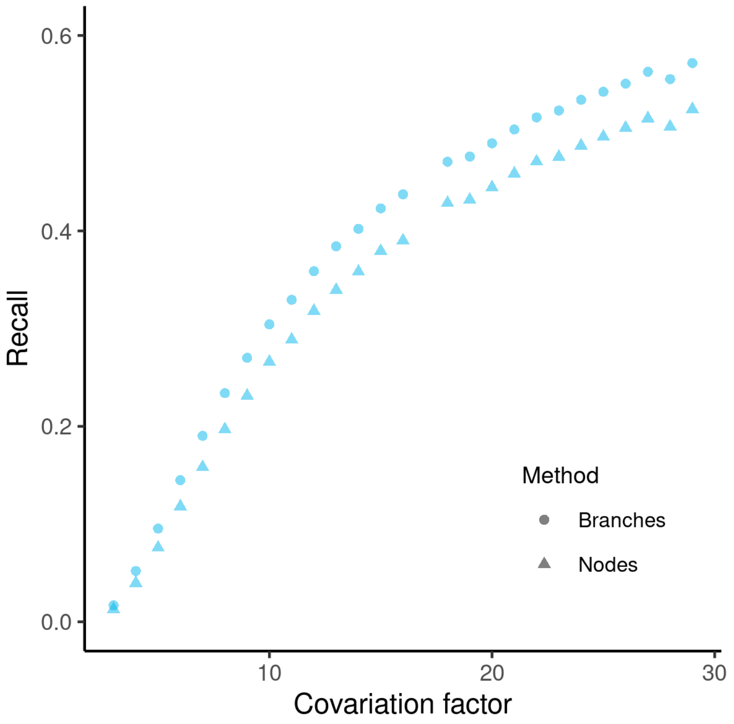

Supplementary Figure 25 Recall values for the Box-Cox model using both the branches and nodes methods at each level of covariation factor parameter.

In addition to the relative performance of the different approaches, we could observe how varying the beta parameters for mutation rate and "branch length" changed the coevolving pair detection rate. Supplementary Figure 26 (for Box-Cox results) shows that detection ability relied more on the "branch length" than overall mutation rate. This implies that greater variation in multiple sequence alignments will enable better detection of coevolving pairs.

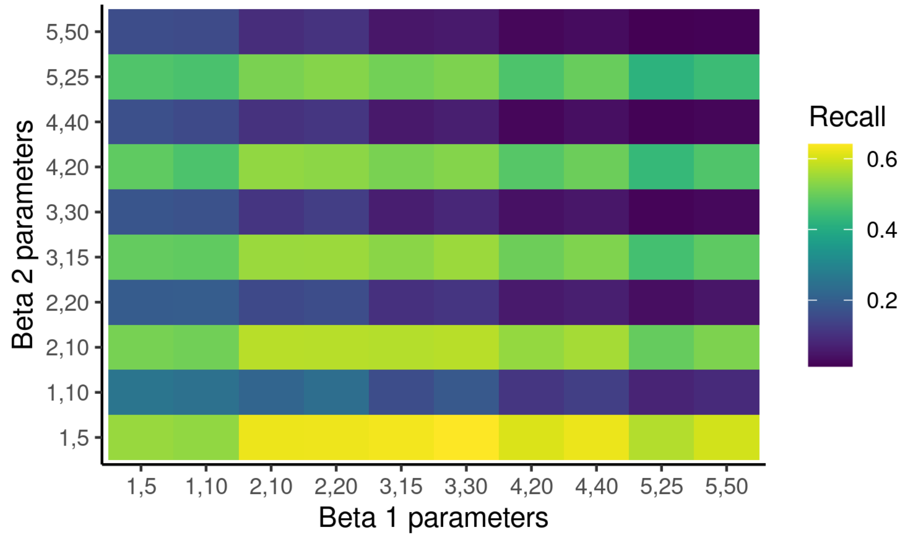

Supplementary Figure 26 Recall for combinations of Beta parameters for "overall mutation rate", Beta 1, and "branch length", Beta 2. Results shown are for the Box-Cox model using the Branches method.

Supplementary Figure 27 shows that depending on the “branch lengths” a covariation multiplicative factor greater than 8 is required to allow detection of 50% of coevolving pairs in this simple simulation. No difference was observed in performance for detection of coevolving pairs at different sequence space distance. As shown in Supplementary Figure 28 whilst there is variation across simulations, no coherent pattern due to sequence distance is observed, with the covariation factor being the main determinant of recall.

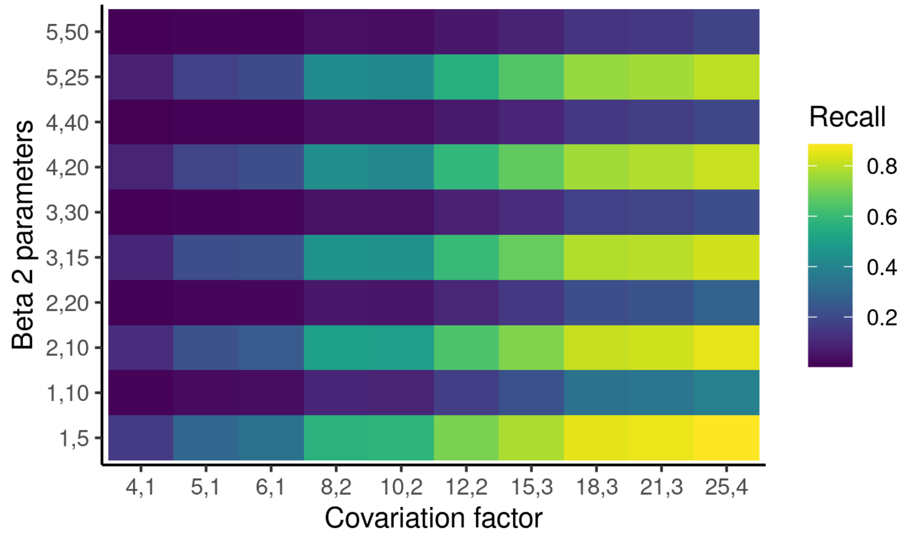

Supplementary Figure 27 Recall for combinations of Beta parameter for "branch length", Beta 2, and the covariation factor parameters. Results shown are for the Box-Cox model using the Branches method.

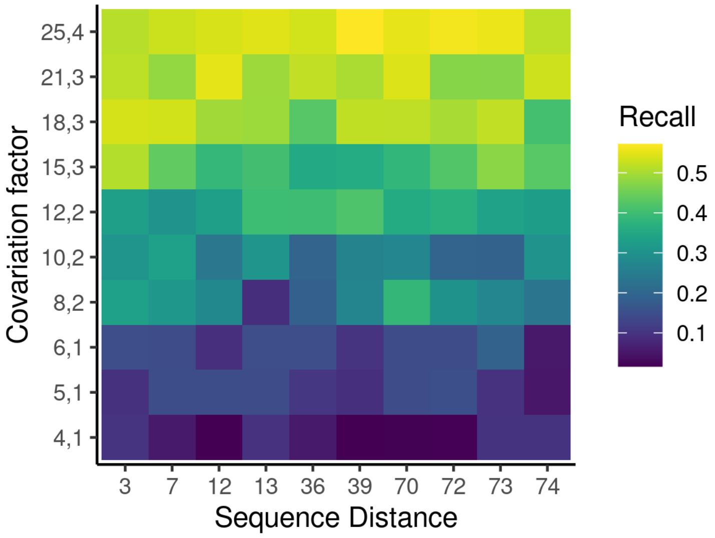

Supplementary Figure 28 Recall for coevolving pairs at different sequence distances versus the covariation factor parameters. Results shown are for the Box-Cox model using the Branches method.

Simulation 4: Multiple coevolving pairs – fixed matrix size

We simulated the different evolutionary rates for each position, and the number of replacements per split as previously explained. We used a Gamma distribution to model multiple covarying pairs. Given *n* positions in the simulation, the number of distinct pairs *m* is $\frac{n\times(n-1)}{2}$. Thus, we drew a sample *s* of size *m* from the Gamma distribution. Each pair was assigned a value from the sample. 1 was added to each of those values because the smallest multiplicative factor used is 1 (i.e. we did not model negative covariation). Finally, the values were rounded to the closest integer (Supplementary Figure 29).

We used 5 different gamma distributions: Gamma(1,1) median 0.693, mode 1; Gamma(1,2) median 1.386, mode 2; Gamma(1,5) median 3.466, mode 2; Gamma(2,1) median 1.678, mode 2; Gamma(5,1) median 4.671, mode 5.

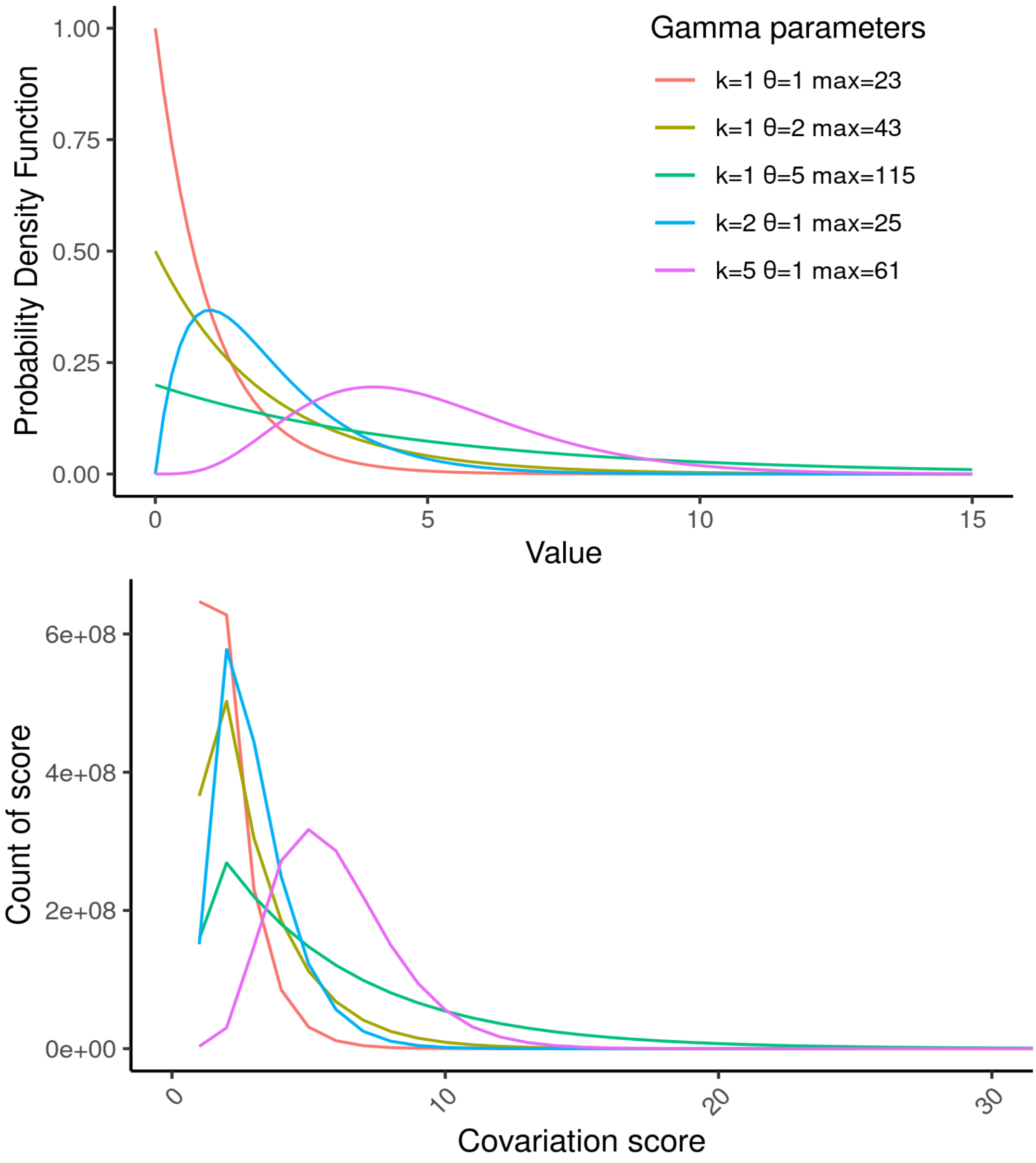

Supplementary Figure 29 Representations of the gamma distributions used to determine the covariation factor for each pair of simulated residues. Above is the shape of the gamma functions used and below is the actual values used, the lack of smoothness due to the fact covariation scores are integers.

Most of the pairs either did not covary (i.e. their multiplicative factor was 1), or had weak covariation (i.e. their multiplicative factor was below 5). According to our previous results, we should not be able to detect many of those weak coevolution pairs.
Each combination of evolutionary rate and proportion of position beta distributions and covariation gamma distributions (500 combinations) was simulated 100 times for a total of 50,000 simulations.

Prediction statistics are reported using two different thresholds for determining a TP (Supplementary Table 6 and Supplementary Table 7). As the fixed pair simulations showed, detection of coevolution when the multiplicative factor is less than 8 was not common. Since most of our gamma distributions have low medians then many pairs will technically be coevolving but at a very low level where detection is not possible. As such, counting any pair with a covariation factor above 1 as a TP is probably not optimal for assessing performance. We have reported results where the covariation threshold had to be above 8 to try and combat this. Unfortunately, this has the side effect of calling anything with a covariation factor of 2 to 8 as a FP even though they do coevolve albeit at a low level (hence the apparent reduction in precision).

Supplementary Table 6 Performance of different combinations of models and methods for identifying coevolving positions in a complex coevolution scenario. Any pair with a multiplicative factor above 1 is considered to be coevolving

Model	Method	Precision	Specificity	Recall	Markedness	Informedness
Linear	Branches	1.000	1.000	0.000	0.160	0.000
Logarithmic	Branches	0.987	1.000	0.000	0.147	0.000
Box-Cox	Branches	0.999	1.000	0.000	0.159	0.000
Linear	Nodes	1.000	1.000	0.000	0.160	0.000
Logarithmic	Nodes	0.987	1.000	0.000	0.147	0.000
Box-Cox	Nodes	0.999	1.000	0.000	0.159	0.000

Supplementary Table 7 Performance of different combinations of models and methods for identifying coevolving positions in a complex coevolution scenario. Any pair with a multiplicative factor above 8 is considered to be coevolving

Model	Method	Precision	Specificity	Recall	Markedness	Informedness
Linear	Branches	0.567	1.000	0.0001	0.490	0.0001
Logarithmic	Branches	0.526	1.000	0.0003	0.450	0.0002
Box-Cox	Branches	0.592	1.000	0.0002	0.515	0.0001
Linear	Nodes	0.570	1.000	0.0001	0.493	0.0001
Logarithmic	Nodes	0.517	1.000	0.0003	0.440	0.0002
Box-Cox	Nodes	0.582	1.000	0.0002	0.506	0.0001

Examining the impact of varying the covariation factors shows that, generally, increasing the covariation multiplicative value increases the proportion of coevolving pairs correctly

identified. The proportions identified using the Box-Cox statistical analysis is shown in Supplementary Figure 30.

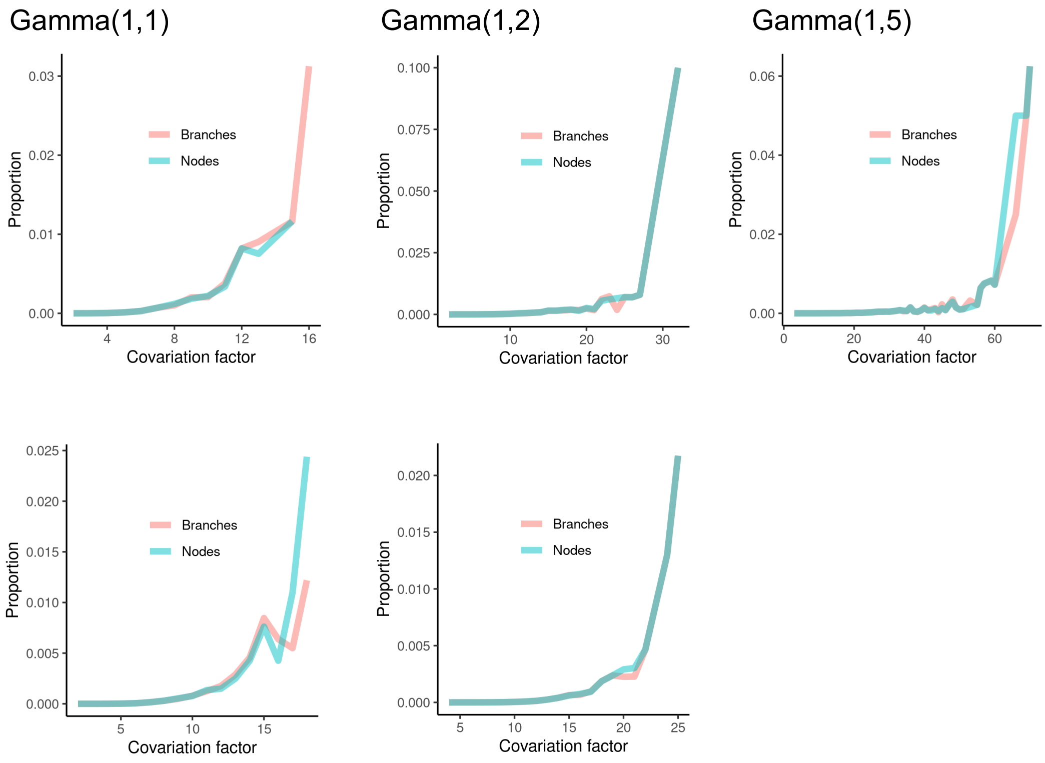

Supplementary Figure 30 Recall of coevolving pairs increases with both methods, using all gamma distributions, increases as the actual assigned covariation factor increases. Results are shown for the Box-Cox model.

We also examined the proportions of changes in a tree that were observed at a single location and how that impacted recall. As shown in Supplementary Figure 31 to detect coevolving pairs there is a level of changes that must occur in both of the residues.

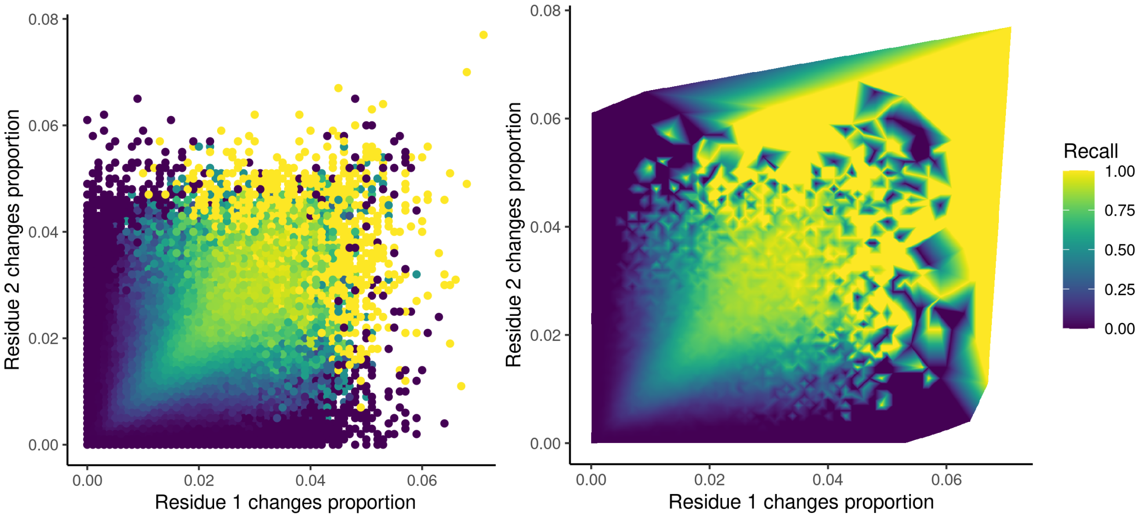

Supplementary Figure 31 The Recall of coevolving pairs shown for the proportion of changes in an alignment that occur at either position. The first panel shows recall for pairs with their proportion of changes rounded to three decimal places, the second panel shows the recall using Akima interpolation. Both panels show results for the Box-Cox model using the branches method.

Simulation 5: Multiple coevolving pairs – variable matrix size

We used the same strategy to simulate variation; however, we analysed the effect of different protein length (i.e. different number of sites), and the effect of different alignment depth (i.e. different number of bifurcations in the tree).

Five different lengths; 50, 100, 200, 400 and 800 as well as 12 differing numbers of tree bifurcations; 50, 100, 200, 400, 800, 1,600, 3,200, 6,400, 12,800, 25,600, 51,200 and 102,400 were simulated. These were combined with the previous evolutionary rate and proportion of position beta distributions and covariation gamma distributions to create 4,800 parameter combinations, each simulated 10 times for a total of 48,000 simulations. We also ran the gamma complete analysis using four different alpha significance thresholds: 0.1, 0.05, 0.01 and 0.001. These would equate to considering coevolving pairs all those that lay outside the 90%, 95%, 99% and 99.9% prediction intervals, respectively. Supplementary Table 8 shows the overall prediction statistics from these simulations.

Supplementary Table 8 Performance of different combinations of models and methods for identifying coevolving positions in a complex coevolution scenario.

Model	Method	Precision	Specificity	Markedness	Informedness
Logarithmic	Branches	0.979	1.000	0.140	0.000
Box-Cox	Branches	0.971	1.000	0.132	0.000
Logarithmic	Nodes	0.979	1.000	0.140	0.000
Box-Cox	Nodes	0.972	1.000	0.131	0.000

The combination of number of events and the length of simulated alignment impacts the precision of coevolving pair prediction. Simulated genes with increased levels of evolutionary events (tree bifurcations) have more accurately detected coevolving pairs. The converse is true for alignment length, with longer sequences showing reduced recall (Supplementary Figure 32).

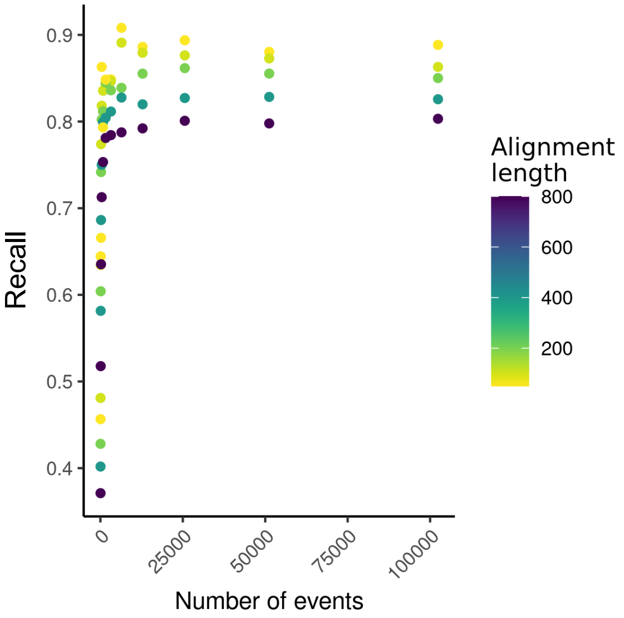

Supplementary Figure 32 Precision for simulations with differing numbers of events and Alignment lengths.

Supplementary Figure 33 shows the precision and specificity metrics for changing alpha, using 8 as the covariation factor threshold (pairs with a multiplicative factor above 8 are considered positives, while the rest are negatives). Specificities are all very high and increase with stricter alpha values, but the precision increase with looser alpha values. It is clear that relaxing alpha increases the probability to identify more TP, but will also increase the probability of predicting FP. Depending on the ratio of positives/negatives, this will affect the precision of the method; i.e. it might have a positive effect in scenarios where there are many positives; however, it would be bad if there were very few.

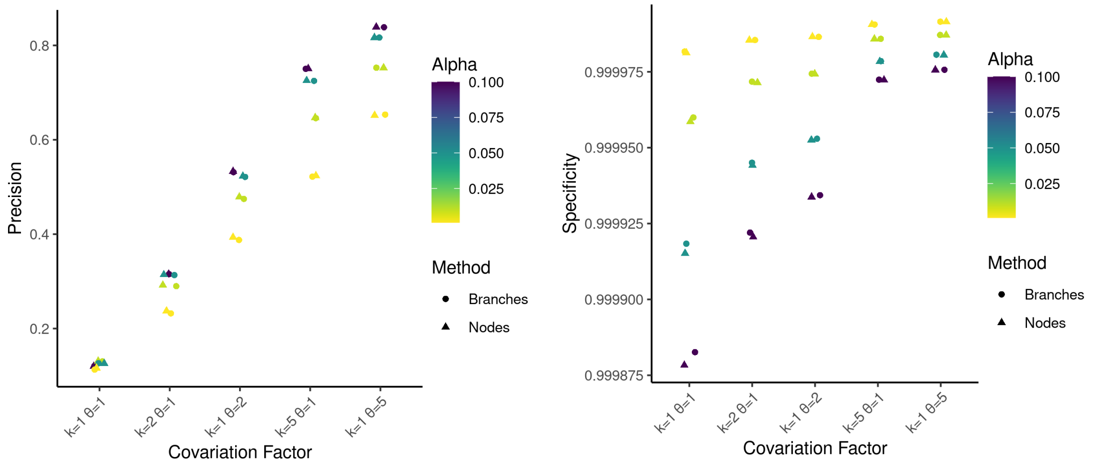

Supplementary Figure 33 Precision (left) and Specificity (right) for Box-Cox model predictions with differing covariation factor parameter distributions when processed at varying alpha value levels.

Branches and nodes overlap

Since the branches and nodes methods potentially have different occurrences of changes in the tree structure taking the overlapping sets of predictions may reduce the number of false positives that may occur from these small differences. To investigate this, we checked the overlapping set of results and compared to the predictions unique to one method or the other in the 10 coevolving pair simulation for the Box-Cox model (Supplementary Table 9).

Supplementary Table 9 Performance of the Box-Cox model with results selected as overlapping or unique to the branches or nodes methods for identifying coevolving positions in the 10 coevolving pair simulation.

Method	Precision	Specificity	Markedness	Informedness
All Branches	0.996	1.000	0.995	0.314
Branches unique	0.983	1.000	0.981	0.042
All Nodes	0.996	1.000	0.994	0.279
Nodes Unique	0.920	1.000	0.918	0.008
Overlap	0.998	1.000	0.997	0.270

Small improvements are observed in Precision and Markedness when taking only overlapping predictions. In addition, the unique predictions for both methods have worse performance in all observed statistics. As we aim to reduce the number of false positives, the overlapping predictions will be the set used for real data.

References

Lui, W. S. (2015). "Prediction Intervals for Poisson Regression." Yet Another Blog in Statistical Computing https://statcompute.wordpress.com/2015/12/20/prediction-intervals-for-poisson-regression/.

Powers, D. M. W. (2011). "Evaluation: from precision, recall and F-measure to ROC, informedness, markedness correlation." J Mach Learn Technol **2**(1): 37-63.

Talavera, D., S. C. Lovell and S. Whelan (2015). "Covariation Is a Poor Measure of Molecular Coevolution." Mol Biol Evol **32**(9): 2456-2468.

Appendix: Parameters used in the simulations

Each simulation was run with a different combination of the below parameters. The simulations with no coevolution did not include any pair covariation factor.

**Positional probability of change**
Beta(1,5)
Beta(1,10)
Beta(2,10)
Beta(2,20)
Beta(3,15)
Beta(3,30)
Beta(4,20)
Beta(4,40)
Beta(5,25)
Beta(5,50)

**Probability of change for each tree bifurcation**[1]
Beta(1,5)
Beta(1,10)
Beta(2,10)
Beta(2,20)
Beta(3,15)
Beta(3,30)
Beta(4,20)
Beta(4,40)
Beta(5,25)
Beta(5,50)

[1] The number of changes per bifurcation is obtained by rounding the value of the probability times the number of sites (i.e. sequence length).

**Covariation multiplicative factor (single coevolving pair)**	**Covariation multiplicative factor distribution (ten coevolving pairs)[1]**	**Covariation multiplicative factor distribution (multiple pairs)[2]**
2	$N(4,1)$	Gamma(1,1)
3	$N(5,1)$	Gamma(1,2)
4	$N(6,1)$	Gamma(1,5)
5	$N(8,2)$	Gamma(2,1)
7	$N(10,2)$	Gamma(5,1)
9	$N(12,2)$	
11	$N(15,3)$	
14	$N(18,3)$	
17	$N(21,3)$	
20	$N(25,4)$	

[1] The multiplicative factors are obtained by rounding up the drawn values.

[2] The multiplicative factors are obtained by rounding up the drawn values plus 1.

**Sequence length**
50
100
200
400
800

Number of tree bifurcations
50
100
200
400
800
1,600
3,200
6,400
12,800
25,600
51,200
102,400

Alpha value for prediction intervals
0.1
0.05
0.01
0.001
